# Recharacterization of the mammalian cytosolic type 2 (*R*)-β-hydroxybutyrate dehydrogenase as 4-oxo-l-proline reductase (EC 1.1.1.104)

**DOI:** 10.1016/j.jbc.2022.101708

**Published:** 2022-02-10

**Authors:** Sebastian Kwiatkowski, Maria Bozko, Michal Zarod, Apolonia Witecka, Kubra Kocdemir, Adam K. Jagielski, Jakub Drozak

**Affiliations:** Department of Metabolic Regulation, Faculty of Biology, Institute of Biochemistry, University of Warsaw, Warsaw, Poland

**Keywords:** 4-oxo-l-proline, *cis*-4-hydroxy-l-proline, *trans*-4-hydroxy-l-proline, short-chain dehydrogenase/reductase superfamily, BDH2, DHRS6, 4-oxo-l-proline reductase, 2,5-DHBA, 2,5-dihydroxybenzoic acid, 2,3-diDHBA, 2,3-dihydro-2,3-dihydroxybenzoic acid, BDH2, type 2 (*R*)-β-hydroxybutyrate dehydrogenase, DMEM, Dulbecco's minimal essential medium, EC, Enzyme Commission, ESI, electrospray ionization, hBDH2, human BDH2, HEK293T, human embryonic kidney 293T cell line, HIFα, hypoxia-inducible factor alpha, l-FDVA, *N*α-(5-fluoro-2,4-dinitrophenyl)-l-valine amide, MS, mass spectrometry, MTT, 3-[4,5-dimethylthiazol-2-yl]-2,5 diphenyl tetrazolium bromide, NCBI, National Center for Biotechnology Information, PLGS, ProteinLynx Global Server, PRODH2, proline dehydrogenase 2, Pyr5C, Δ1-pyrroline-3-hydroxy-5-carboxylate, Q-TOF, quadrupole time of flight, rBDH2, rat BDH2, RP–HPLC, reversed-phase HPLC

## Abstract

Early studies revealed that chicken embryos incubated with a rare analog of l-proline, 4-oxo-l-proline, showed increased levels of the metabolite 4-hydroxy-l-proline. In 1962, 4-oxo-l-proline reductase, an enzyme responsible for the reduction of 4-oxo-l-proline, was partially purified from rabbit kidneys and characterized biochemically. However, only recently was the molecular identity of this enzyme solved. Here, we report the purification from rat kidneys, identification, and biochemical characterization of 4-oxo-l-proline reductase. Following mass spectrometry analysis of the purified protein preparation, the previously annotated mammalian cytosolic type 2 (*R*)-β-hydroxybutyrate dehydrogenase (BDH2) emerged as the only candidate for the reductase. We subsequently expressed rat and human BDH2 in *Escherichia coli*, then purified it, and showed that it catalyzed the reversible reduction of 4-oxo-l-proline to *cis*-4-hydroxy-l-proline *via* chromatographic and tandem mass spectrometry analysis. Specificity studies with an array of compounds carried out on both enzymes showed that 4-oxo-l-proline was the best substrate, and the human enzyme acted with 12,500-fold higher catalytic efficiency on 4-oxo-l-proline than on (*R*)-β-hydroxybutyrate. In addition, human embryonic kidney 293T (HEK293T) cells efficiently metabolized 4-oxo-l-proline to *cis*-4-hydroxy-l-proline, whereas HEK293T BDH2 KO cells were incapable of producing *cis*-4-hydroxy-l-proline. Both WT and KO HEK293T cells also produced *trans*-4-hydroxy-l-proline in the presence of 4-oxo-l-proline, suggesting that the latter compound might interfere with the *trans*-4-hydroxy-l-proline breakdown in human cells. We conclude that BDH2 is a mammalian 4-oxo-l-proline reductase that converts 4-oxo-l-proline to *cis*-4-hydroxy-l-proline and not to *trans*-4-hydroxy-l-proline, as originally thought. We also hypothesize that this enzyme may be a potential source of *cis*-4-hydroxy-l-proline in mammalian tissues.

4-Oxo-l-proline is a poorly studied analog of l-proline (Fig. 1). The only known natural compound containing a 4-oxo-l-proline moiety is the antibiotic X-type actinomycin produced by *Streptomyces antibioticus* (1). Nevertheless, 4-oxo-l-proline has been detected occasionally in various biological samples, including extracts of human embryonic kidney 293T (HEK293T) cells (2) and the blood samples of type 2 diabetes patients treated with metformin, sulphonylurea, or both drugs combined (3). Unfortunately, the exact source and metabolic routes of 4-oxo-l-proline *in vivo* have never been addressed appropriately, and our knowledge of its physiological significance is still sparse. Interestingly, in the late 1950s, Mitoma *et al.* (4) showed that incubating 4-oxo-l-proline with chick embryos increased the free 4-hydroxy-l-proline content in their developing bodies, suggesting that 4-oxo-l-proline might be enzymatically reduced *in vivo*. Later, 4-oxo-l-proline reductase (Enzyme Commission [EC] number: EC 1.1.1.104), a cytosolic and NADH-dependent enzyme converting 4-oxo-l-proline into 4-hydroxy-l-proline (Fig. 1), was partially purified from rabbit kidney and characterized (5), but its molecular identity and biochemical properties have remained unknown so far.

**Figure 1:**
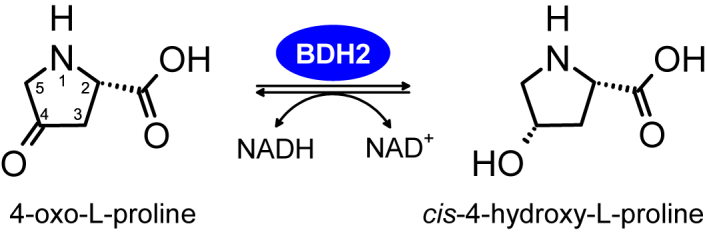
**Scheme of the reaction catalyzed by BDH2 as a 4-oxo-l-proline reductase.** BDH2 also acts as a *cis*-4-OH-l-proline dehydrogenase in the presence of the NAD^+^*in vitro*. The biological relevance of the reverse reaction remains to be explored. BDH2, type 2 (*R*)-β-hydroxybutyrate dehydrogenase.

4-Hydroxy-l-proline can exist as *trans* and *cis* diastereomers. In mammals, the former is the product of post-translational modification of l-proline residues present in certain proteins, such as collagen, elastin, protein kinase B, complement protein C1q, or hypoxia-inducible factor alpha (HIFα) proteins (6, 7). *trans*-Hydroxylation of peptidic proline is catalyzed by prolyl 4-hydroxylase (EC 1.14.11.29), also known as procollagen-proline 4-dioxygenase (EC 1.14.11.2) (7). This particular post-translational modification confers structural stability on the triple helix of collagen but may also be a signal for protein degradation, as in the case of HIF-1α transcription factor. Upon hydroxylation of specific proline residues, HIF-1α protein is recognized by the ubiquitin E3 ligase, polyubiquitinated, and directed to the proteasome for proteolysis (7). However, *cis*-4-hydroxy-l-proline is currently considered absent from mammalian tissues and naturally occurs only in some species of bacteria, fungi, and plants (8, 9, 10). Interestingly, *cis*-4-hydroxy-l-proline was shown to incorporate into collagens, preventing the correct hydroxylation of l-proline residues and the assembly of the triple helix (11). This may lead to intracellular accumulation of misfolded collagen chains and subsequent inhibition of proliferation of normal and malignant tumor cells (12, 13). Therefore, the *cis* diastereomer was evaluated as a potential antifibrotic and anticancer drug in an earlier study (14).

In the current work, we report the identification of mammalian 4-oxo-l-proline reductase (EC 1.1.1.104) as a 3-hydroxybutyrate dehydrogenase type 2 (BDH2 [(*R*)-β-hydroxybutyrate dehydrogenase type 2], DHRS6, Fig. 2). BDH2 was previously suggested to act as a cytosolic BDH involved in ketone body utilization (15) or to catalyze the synthesis of 2,5-dihydroxybenzoic acid (2,5-DHBA; gentisic acid), a putative mammalian siderophore (16). We characterized this enzyme biochemically and showed that it catalyzes the reversible conversion of 4-oxo-l-proline to *cis*-4-hydroxy-l-proline, with distinct substrate specificity and catalytic efficiency. BDH2 is, therefore, the first mammalian enzyme capable of yielding *cis*-4-hydroxy-l-proline, a metabolite thought to only be of microbial and plant origin (8, 9). We also revealed that intact HEK293T cells efficiently metabolize 4-oxo-l-proline to *cis*-4-hydroxy-l-proline. This reaction is absent in *Bdh2*-deficient HEK293T cells, indicating that the activity of BDH2 might be considered as a potential source of *cis*-4-hydroxy-l-proline in various mammalian tissues. Finally, we showed that 4-oxo-l-proline exerted cytotoxic effects on HEK293T cells and hypothesized that the physiological role of BDH2 might be to eliminate this toxic metabolite.

**Figure 2:**
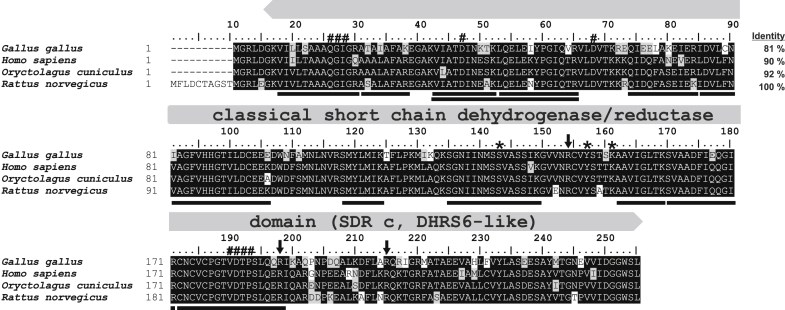
**Amino acid sequence alignment of rat BDH2 (rBDH2) protein with its orthologs.** Sequences of rat (*Rattus norvegicus*, NP_001099943.1), human (*Homo sapiens*, NP_064524.3), rabbit (*Oryctolagus cuniculus*, XP_002717250.1), and chicken (*Gallus gallus*, XP_015141101.1) protein were obtained from the National Center for Biotechnology Information protein database. The percentage of amino acid identities with rBDH2 protein is given in the *upper right*. The label above the alignment indicates the characteristic one-domain architecture of the enzyme. Amino acid residues interacting with NAD(H) are indicated by *hashes*, whereas *arrows* show arginine residues coordinating the carboxylic group of a substrate. *Asterisks* mark the key catalytic residues (15). The peptides identified by mass spectrometry in the protein purified from rat kidneys are underlined in the rat sequence, and several peptides covering similar though shorter sequences have been omitted. The level of residues conservation is indicated by *black* (100%) and *gray* (50% and more) background. BDH2, type 2 (*R*)-β-hydroxybutyrate dehydrogenase.

## Results

### Purification and identification of rat 4-oxo-l-proline reductase

**Figure 3:**
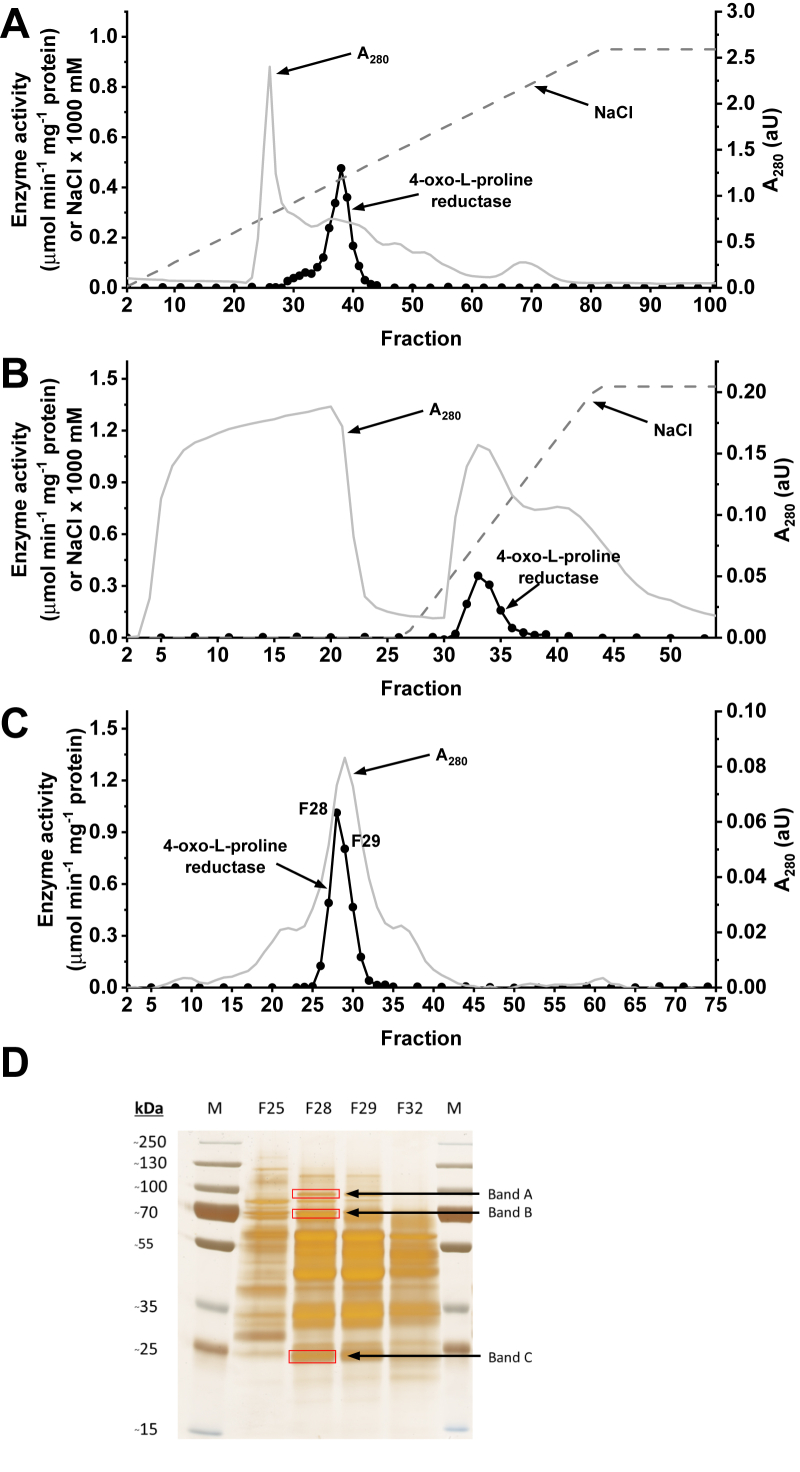
**Purification of the rat 4-oxo-****l****-proline reductase.** The enzyme was purified by column chromatography on (*A*) Q Sepharose, (*B*) HiScreen Blue FF, and (*C*) Superdex 200 16/60 HiLoad as described in the “Experimental procedures” section. Resulted fractions were tested for activity of 4-oxo-l-proline reductase. *D*, the indicated fractions from the Superdex 200 column were analyzed by SDS-PAGE, and the gel was silver stained (30). Indicated bands, which staining intensity followed the enzymatic activity, were cut out from the gel and analyzed by mass spectrometry.

At various stages of the purification process, 4-oxo-l-proline reductase was assayed spectrophotometrically by measuring the rate of 4-oxo-l-proline reduction with concomitant oxidation of NADH to NAD^+^. The enzyme was purified from rat kidneys about 280-fold by a three-step column chromatography involving anion-exchange chromatography on Q Sepharose FF resin, affinity chromatography on HiScreen Blue FF column, and gel filtration on Superdex 200 16/60 HiLoad column. Enzyme activity corresponded with one peak throughout the purification process (Fig. 3). The yield of the purification was 25% based on total recovered activity (Table 1).

**Table 1:** Purification of 4-oxo-l-proline reductase from rat kidneys

Fraction	Volume	Total protein	Total activity	Specific activity	Purification	Yield
	ml	mg	μmol.min^−1^	μmol.min^−1^.mg^−1^	X-fold	%
10–20% PEG	120	2344	13.7	0.006	1	100
Q Sepharose FF	20	148	7.1	0.048	8	52
HiScreen Blue FF	12	17	3.1	0.18	31	22
Superdex 200 16/60 HiLoad	4.5	2.1	3.5	1.65	283	25

SDS-PAGE analysis revealed three polypeptides of about 90, 70, and 25 kDa (*cf.* Fig. 3*D*) that coeluted with the enzyme activity in the fractions derived from the Superdex 200 purification step. All three bands were excised from the gel, digested with trypsin, and the resulting peptides were analyzed by tandem mass spectrometry (MS; quadrupole time of flight [Q-TOF]). The sequences of the identified peptides were then compared with the rat reference proteome from the National Center for Biotechnology Information (NCBI) protein database (Table 2). Surprisingly, the MS sequence data revealed proteins of known function only. However, we hypothesized that the identified BDH2 protein might be a functional 4-oxo-l-proline reductase. MS analysis found 26 peptides (Fig. 2) that covered ≈54% of the rat BDH2 (rBDH2) sequence. To exclude the possibility of missing any potential reductases because of poor extraction of tryptic peptides from the polyacrylamide gel, we also performed tandem MS identification of all proteins present in the most active fractions eluting from the Superdex 200 column (F28 and F29, *cf.* Table 2). Again, BDH2 was found as the only dehydrogenase of unclear biochemical function that could be a reasonable candidate for the rat 4-oxo-l-proline reductase. This conclusion was based mainly on the following findings: (i) the native molecular weight of 4-oxo-l-proline reductase was in the range of 80 to 100 kDa, based on gel filtration (not shown), which was close to the value previously reported for recombinant BDH2 (≈117 kDa) (15), (ii) besides the well-characterized lactate and isocitrate dehydrogenases, BDH2 was the only cytosolic dehydrogenase identified in the most active fraction (F28) eluting from the Superdex 200 column (*cf.* Table 2), and (iii) BDH2 was present as a predominant protein in band C that coeluted with the enzyme activity (*cf.* Fig. 3*D*).

**Table 2:** Proteins identified in fraction 28 from Superdex 200 purification step and gel bands submitted to trypsin digestion and MS/MS analysis

Source of protein	Protein name	NCBI protein accession number	PLGS score^a^	Peptides	Molecular weight (Da)	Coverage (%)
Gel filtration fraction (F28)	60 kDa heat shock protein	NP_071565.2	65,807	69	60,917	80
	l-lactate dehydrogenase B	NP_001303262.1	20,895	21	37,307	50
	Ornithine aminotransferase	NP_071966.1	20,353	27	48,302	64
	Isocitrate dehydrogenase	NP_113698.1	18,164	30	46,704	62
	Serine hydroxymethyltransferase	NP_001008323.1	8295	23	55,729	56
	3-Hydroxybutyrate dehydrogenase type 2	NP_001099943.1	6977	10	27,660	33
	Protein disulfide isomerase A3	NP_059015.1	6086	20	56,553	27
	Hemoglobin alpha 2	NP_001007723.1	5306	5	15,274	44
	Dihydrolipoyl dehydrogenase	NP_955417.1	5272	8	54,004	26
	Ezrin	NP_062230.1	5128	18	69,347	24
Gel band A	Dimethylglycine Dehydrogenase	NP_620802.2	10,426	58	95,917	57
	Ezrin	NP_062230.1	1280	10	69,347	20
Gel band B	Heat shock cognate 71 kDa protein	NP_077327.1	17,565	56	70,827	61
	GMP synthase glutamine hydrolyzing	NP_001019925.1	2312	22	76,708	47
	Plastin 3	NP_112346.1	1911	17	70,704	38
	Heat shock protein 75 kDa	NP_001034090.1	863	13	80,410	23
Gel band C	3-Hydroxybutyrate dehydrogenase type 2	NP_001099943.1	16,517	26	27,660	54
	Ornithine aminotransferase	NP_071966.1	654	3	48,302	17

Identified proteins are listed according to their score as calculated using PLGS software. The molecular weight, sequence coverage, and the number of distinct peptides assigned for each protein are also indicated. Occasional peptide hits corresponding to keratins have not been included.

The complete list of identified proteins and assigned peptides is shown in File S1.

a PLGS score is calculated by the PLGS (version 2.4) software using a Monte Carlo algorithm to analyze all acquired mass spectral data and is a statistical measure of the accuracy of assignation. A higher score implies greater confidence in protein identity.

### Human and rBDH2 catalyze the reversible reduction of 4-oxo-l-proline to *cis*-4-hydroxy-l-proline

**Figure 4:**
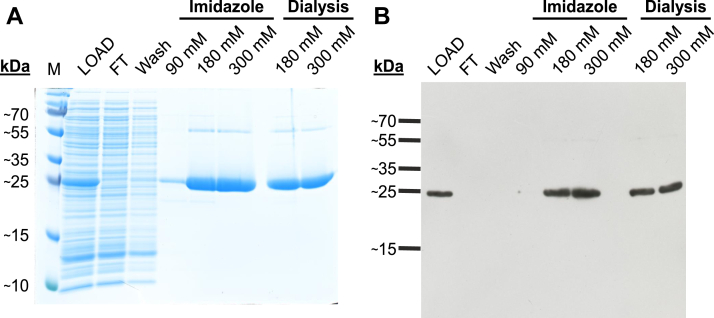
**SDS-PAGEandWestern blot analysis of recombinant rat BDH2 (rBDH2) purification.** Lysate of *Escherichia coli* BL21 culture (LOAD) was applied to HisTrap FF Crude column, and flow-through (FT) was collected. The column was washed with a buffer without imidazole (wash). Retained proteins were eluted by applying the buffer with indicated concentrations of imidazole. To remove imidazole, fractions of 180 and 300 mM were subjected to dialysis. The purification process was analyzed by SDS-PAGE (*A*), whereas the presence of recombinant rBDH2 protein was verified by Western blot analysis (*B*) using an antibody against the His_6_ tag. Analogous results were obtained for human BDH2. The purity of both recombinant proteins was above 95%. M, prestained protein marker. BDH2, type 2 (*R*)-β-hydroxybutyrate dehydrogenase.

**Figure 5:**
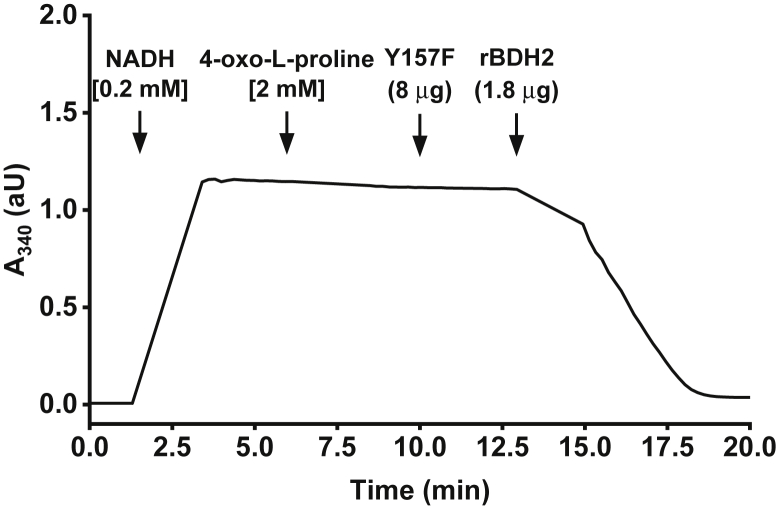
**Test of the enzymatic activity of the purified recombinant WT (rat BDH2 [rBDH2]) and mutated (Y157F) rBDH2.** The enzyme activity was followed spectrophotometrically by measuring the conversion of NADH into NAD^+^ (λ = 340 nm). The reaction was performed with 8 μg of Y157F protein as described in the “Experimental procedures” section. The addition of 1.8 μg of the WT rBDH2 resulted in the complete oxidation of the NADH as indicated by the change in the absorbance (absorbance at 340 nm). A similar result was obtained for the mutated human form of BDH2 (Y147F). The figure shows the results of a single representative assay. The results for control reactions carried out in the absence of 4-oxo-l-proline are shown in Fig. S1. BDH2, type 2 (*R*)-β-hydroxybutyrate dehydrogenase.

To verify that the rBDH2 was a 4-oxo-l-proline reductase, it was overexpressed as a fusion protein with the *N*-terminal His_6_ tag in a bacterial expression system. The recombinant enzyme was purified (Fig. 4) and shown to catalyze the reduction of 4-oxo-l-proline in the presence of NADH (Figs. 5 and S1). Although the rBDH2 and human BDH2 (hBDH2) showed 90% identity in their amino acid sequences (*cf.* Fig. 2), implying similar enzymatic properties, the hBDH2 was expressed similarly to compare its activity.

To further confirm that the observed activity resulted from BDH2 and not from impurities that might copurify with the recombinant proteins, mutated forms of both orthologs were also produced (Y157F and Y147F for rat and human, respectively). The Y147 residue and its rat equivalent (Y157) were chosen for mutagenic studies since Y147 was shown to be a catalytic residue interacting with the carbonyl group of (*R*)-β-hydroxybutyrate, a prototypic BDH2 substrate, and its substitution by F147 completely abolished the activity of BDH2 (15). These mutants were catalytically inactive with 4-oxo-l-proline and NADH as substrates (*cf.* Figs. 2 and 5), confirming that BDH2 had the 4-oxo-l-proline reductase activity.

Because of the high amino acid sequence and structural similarities to various bacterial hydroxybutyrate dehydrogenases, BDH2 was previously shown to be involved in the ketone body (*R*)-β-hydroxybutyrate metabolism (15). Furthermore, BDH2 has also been proposed to catalyze the synthesis of 2,5-DHBA (gentisic acid), a putative mammalian siderophore (16). These reports led us to verify the substrate specificity of BDH2 with biologically relevant substrates, including oxidizing (*R*)-β-hydroxybutyrate, l-threonine, l-serine, l-homoserine, and the two isomers of 4-hydroxy-l-proline and reducing 5-oxo-l-proline (l-pyroglutamic acid) and acetoacetate; the latter is a product of the postulated BDH activity of BDH2.

Out of all tested compounds, only 4-oxo-l-proline, *cis*-4-hydroxy-l-proline, and, albeit to a much lesser extent, (*R*)-β-hydroxybutyrate and l-threonine were substrates for BDH2 (Table 3). Importantly, *trans*-4-hydroxy-l-proline, which is thought to be the product of this reductase activity so far (5), was not oxidized to 4-oxo-l-proline in the reverse reaction, indicating a stereospecific preference of the enzyme for *cis*-4-hydroxy-l-proline. Furthermore, a negligible specific activity toward (*R*)-β-hydroxybutyrate (0.04 ± 0.00 and 0.05 ± 0.00 μmol min^−1^ mg^−1^ protein for rBDH2 and hBDH2, respectively) comparing with the results for *cis*-4-hydroxy-l-proline (15.7 ± 0.4 and 21.2 ± 0.4 for rBDH2 and hBDH2, respectively) and 4-oxo-l-proline (22.9 ± 1.3 and 31.4 ± 0.8 for rBDH2 and hBDH2, respectively) indicate that (*R*)-β-hydroxybutyrate is unlikely to be a physiological substrate for BDH2. Similarly, no more than just a negligible activity of BDH2 was detected toward l-threonine (0.001 ± 0.000 and 0.002 ± 0.000 μmol min^−1^ mg^−1^ protein for rBDH2 and hBDH2, respectively). Finally, l-serine and l-homoserine were not the substrates for the enzyme (*cf.* Table 3).

**Table 3:** Substrate specificity of the recombinant rBDH2 and hBDH2

Structure	Substrate	hBDH2	rBDH2
		μmol min^−1^ mg^−1^	μmol min^−1^ mg^−1^
	2 mM 4-oxo-l-proline	31.4 ± 0.8	22.9 ± 1.3
	2 mM 5-oxo-l-proline	ND	ND
	2 mM *trans*-4-OH-l-proline	ND	ND
	2 mM *cis*-4-OH-l-proline	21.2 ± 0.4	15.7 ± 0.4
	2 mM acetylacetate	ND	ND
	2 mM (*R*)-β-hydroxybutyrate	0.011 ± 0.000	0.007 ± 0.000
	50 mM (*R*)-β-hydroxybutyrate^a^	0.050 ± 0.003	0.040 ± 0.002
	50 mM l-threonine	0.002 ± 0.000	0.001 ± 0.000
	50 mM l-serine	ND	ND
	50 mM l-homoserine	ND	ND

Activity assays were performed with the use of 2 μg (4-oxo-l-proline and *cis*-4-hydroxy-l-proline), 30 μg ((*R*)-β-hydroxybutyrate), or 40 μg (l-serine, l-homoserine, and l-threonine) of recombinant BDH2 proteins in the presence of 0.2 mM NADH or 1 mM NAD^+^ when necessary and indicated concentration of the potential substrate. Values are the means ± SD (error bars) of three independent experiments.

Abbreviation: ND, not detectable.

a 50 mM concentration of (*R*)-β-hydroxybutyrate is considered to be saturating (15).

**Figure 6:**
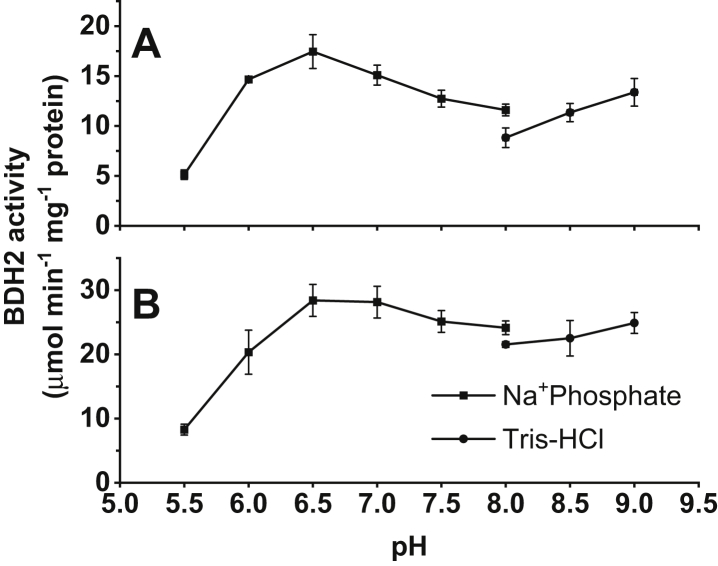
**Effect of the pH on the activity of the recombinant rat and human BDH2.** The activity of (*A*) rat and (*B*) human enzyme was followed spectrophotometrically by measuring the conversion of NADH into NAD^+^ (λ = 340 nm). The reaction was performed with 2 µg of enzyme proteins at different pH values of the reaction mixture as described in the “Experimental procedures” section. Eighty millimolar sodium phosphate buffer was used for the lower pH values, and 50 mM Tris–HCl was used for higher pH values. The activity of both recombinant enzymes was slightly lower in the Tris–HCl buffer compared with sodium phosphate buffer at pH = 8.0. Values are the means ± SD (error bars) of three independent experiments. When an error bar is not visible, the error is smaller than the width of the line. BDH2, type 2 (*R*)-β-hydroxybutyrate dehydrogenase.

It was previously reported that the optimum pH value for the reaction catalyzed by 4-oxo-l-proline reductase is about 6.5 (5). To verify this information, we determined the pH range of the reductase activity (Fig. 6). Interestingly, BDH2 remained catalytically active in a broad pH spectrum (from 5.5 through 9.0). Moreover, the pH optimum for the rat enzyme was more evident at 6.5, whereas the human enzyme exhibited the highest activity in the pH range from 6.5 to 7.0, with only a slight decrease in higher pH values. Notably, the activity of BDH2 orthologs toward *cis*-4-hydroxy-l-proline and other substrates in a reduced form was only detected in the presence of 1 mM NAD^+^ and at pH 9.0, suggesting that the reaction equilibrium is strongly shifted toward the reduction of substrates. These results indicate that BDH2 is most likely the same 4-oxo-l-proline-reducing enzyme studied by Smith and Mitoma (5).

The kinetic properties of the recombinant BDH2 proteins were investigated in detail using homogenous recombinant proteins and are compared in Table 4, and the analyses are shown in Fig. S2. Both enzymes followed the Michaelis–Menten model of enzyme kinetics. The *K*_*M*_ values for 4-oxo-l-proline obtained in our investigation (*K*_*M*_ ≈ 0.4–0.5 mM) were comparable to those determined for the partially purified rabbit enzyme (*K*_*M*_ ≈ 0.6 mM) (5). In contrast, the *K*_*M*_ values of the recombinant hBDH2 and rBDH2 for NADH could not be accurately determined in the current study because the sensitivity of the spectrophotometric assay was limited to ≈2 μM. However, they were roughly estimated at ≈3 μM, much lower than reported by Smith and Mitoma (5) (*K*_*M*_ ≈ 840 μM) (Fig. S3). Finally, both hBDH2 and rBDH2 revealed a minimal affinity toward (*R*)-β-hydroxybutyrate, with the *K*_*M*_ values in the range of 6 to 10 mM (*cf.* Table 4 and Fig. S2).

**Table 4:** Kinetic properties of rBDH2 and hBDH2 proteins

Substrate	rBDH2			hBDH2		
	*V*_max_	*K*_*M*_	*k*_cat_^a^	*V*_max_	*K*_*M*_	*k*_cat_^a^
	μmol min^−1^ mg^−1^	μM	s^−1^	μmol min^−1^ mg^−1^	μM	s^−1^
4-oxo-l-proline	28.29 ± 0.42	387.49 ± 7.03	14 ± 0.21	41.21 ± 0.89	483.03 ± 0.01	19.73 ± 0.43
(*R*)-β-hydroxybutyrate	0.04 ± 0.00	10,232 ± 331	0.02 ± 0.00	0.04 ± 0.00	6,152 ± 365	0.02 ± 0.00

Kinetic properties were determined with the use of purified recombinant *N*-terminal His_6_-tagged BDH2 proteins. Determinations for 4-oxo-l-proline were performed with 2 μg of the enzyme preparations in the presence of 0.2 mM NADH and variable concentrations of 4-oxo-l-proline. The *K*_*M*_ value for NADH was roughly estimated as ≈3 μM. The measurements for (*R*)-β-hydroxybutyrate were performed with 30 μg of the enzyme preparations in the presence of 1 mM NAD^+^. Values are the means ± SD (error bars) of three independent experiments.

a Calculated for the His_6_-tagged recombinant enzymes with molecular weight = 29,703 and 28,722 Da for the rat and human enzymes, respectively.

### Evidence for *cis*-4-hydroxy-l-proline as the product of BDH2 activity

**Figure 7:**
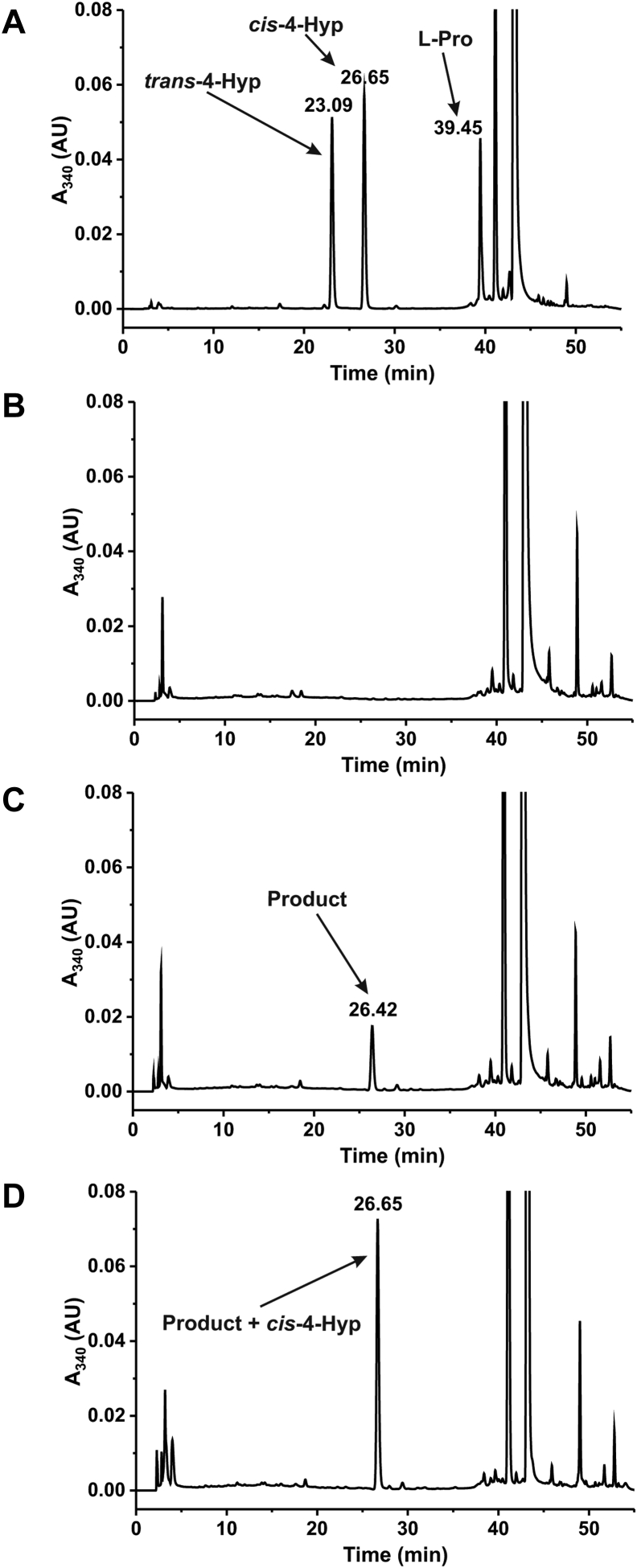
**RP-HPLC analysis of the product formed by human BDH2 protein.** Shown are chromatograms (*A*) of a standard mixture of *cis*-4-hydroxy-l-proline (*cis*-4-Hyp), *trans*-4-hydroxy-l-proline (*trans*-4-Hyp), and l-proline (0.5 nmol) after derivatization with L-FDVA. *B*, of deproteinized reaction mixtures obtained during incubation of homogenous recombinant human protein (2 μg) with 2 mM 4-oxo-l-proline and 0.2 mM NADH for 0 min or (*C*) 15 min as well as (*D*) following the supplementation of the former deproteinized reaction mixture with 0.5 nmol of *cis*-4-hydroxy-l-proline standard. The identity of all indicated compounds was confirmed by tandem mass spectrometry. The sample processing and chromatographic conditions are described under “Experimental procedures” section. BDH2, type 2 (*R*)-β-hydroxybutyrate dehydrogenase; L-FDVA, *N*α-(5-fluoro-2,4-dinitrophenyl)-l-valine amide; RP-HPLC, reversed-phase HPLC.

**Figure 8:**
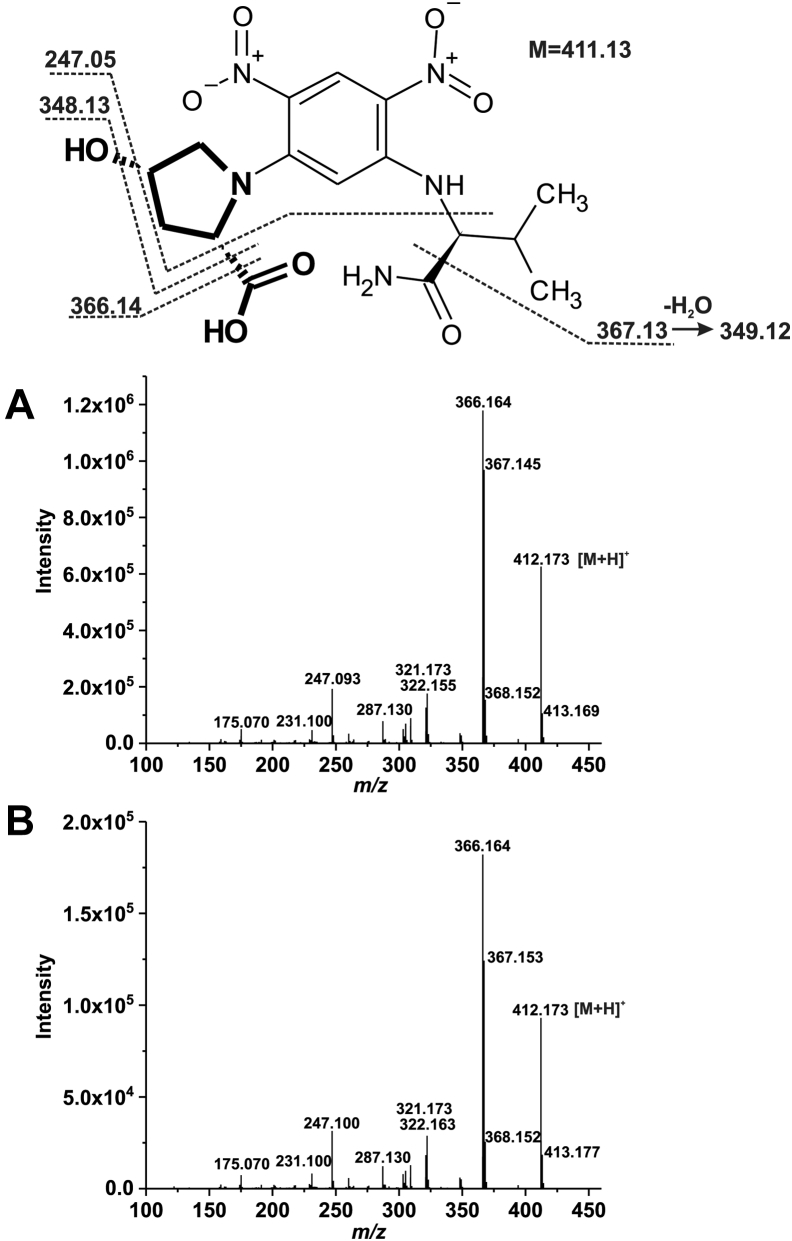
**Q-TOF fragmentation spectra of L-FDVA derivatives of *cis*-4-hydroxy-****l****-proline and the product formed by human BDH2 protein.** The homogenous recombinant human enzyme was incubated for 15 min with 2 mM 4-oxo-l-proline and 0.2 mM NADH, and the progress of the reaction was followed spectrophotometrically at λ = 340 nm. The product was then derivatized with L-FDVA, chromatographed on a reversed-phase C18 column, and analyzed by tandem mass spectrometry. Mass spectra, covering the mass range *m/z* 100 to 450, (*A*) of commercial *cis*-4-hydroxy-l-proline derivatized with L-FDVA and (*B*) the product biocatalyzed by human BDH2 enzyme were acquired. The structure of the L-FDVA derivative of *cis*-4-hydroxy-l-proline (in *bold*) and the assignments of some of its fragment ions are also shown. BDH2, type 2 (*R*)-β-hydroxybutyrate dehydrogenase; L-FDVA, *N*α-(5-fluoro-2,4-dinitrophenyl)-l-valine amide; Q-TOF, quadrupole time of flight.

Until now, the reaction catalyzed by 4-oxo-l-proline reductase was thought to produce *trans*-4-hydroxy-l-proline, but this view was based on the results on just an elementary chromatographic analysis (5). To determine the stereoconfiguration of 4-hydroxy-l-proline biocatalyzed by recombinant hBDH2, the perchloric acid precipitated the proteins, and the clarified supernatant was subjected to precolumn chiral derivatization with *N*α-(5-fluoro-2,4-dinitrophenyl)-l-valine amide (l-FDVA), followed by reversed-phase HPLC (RP–HPLC) and mass spectrometric analysis (17). Chromatographic analysis of the derivatized product revealed its comigration with a commercial standard of *cis*-4-hydroxy-l-proline derivatized identically (Fig. 7). The addition of authentic *cis*-4-hydroxy-l-proline to the reaction mixture containing the biocatalyzed product before its derivatization resulted in a selective increase in the peak area corresponding to the derivatized product without skewing the peak symmetry, supporting the identity as the *cis*-4-hydroxy-l-proline. Analysis of the product by electrospray ionization Q-TOF (ESI-Q-TOF) MS indicated a protonated parent molecular ion with *m/z* 412 consistent with an L-FDVA derivative of *cis*-4-hydroxy-l-proline (17). The tandem MS analysis of this ion revealed a fragmentation pattern for the biocatalyzed product identical to that of the commercial standard of *cis*-4-hydroxy-l-proline derivatized the same (Fig. 8). Notably, the Q-TOF fragmentation pattern of a commercial standard of the derivatized *trans*-4-hydroxy-l-proline was different from that of the cognate *cis*-4-hydroxy-l-proline, confirming that recombinant BDH2 catalyzes the conversion of 4-oxo-l-proline to *cis*-4-hydroxy-l-proline (Fig. S4).

These results confirm that BDH2 catalyzes the formation only of *cis*-4-hydroxy-l-proline, which has been considered as a microbial and a plant metabolite (8, 9) and nonphysiological compound in mammalians (10).

### HEK293T cells metabolize 4-oxo-l-proline into *cis*-4-hydroxy-l-proline

**Figure 9:**
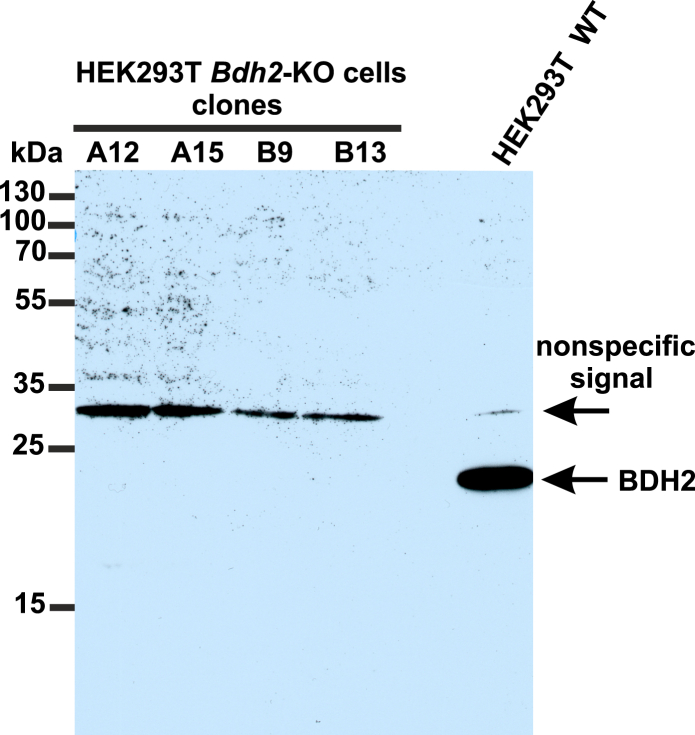
**Western blot analysis of BDH2 expression in four *Bdh2*-deficient HEK293T clonal cell lines (*Bdh2*-KO) and WT HEK293T cells (WT).** The KO clonal cell lines (A12, A15, B9, and B13) were generated by the CRISPR–Cas9 gene-inactivation procedure. The Western blot analysis was carried out using 40 μg of the cell lysate protein, a primary rabbit antibody against the human BDH2 (catalog no.: PA5-44760; Invitrogen), and a horseradish peroxidase–conjugated goat anti-rabbit secondary antibody. The secondary antibody was detected by measuring enhanced chemiluminescence. The presence of a nonspecific signal (≈30 kDa) is in agreement with the specification of the primary antibody. BDH2, type 2 (*R*)-β-hydroxybutyrate dehydrogenase; HEK293T, human embryonic kidney 293T cell line.

To verify whether 4-oxo-l-proline is converted into *cis*-4-hydroxy-l-proline in whole human cells, we initially investigated its metabolism in the intact HEK293T cells that express the BDH2 enzyme (Fig. 9). The cells or cell-free culture medium (control) were incubated with or without 1 mM 4-oxo-l-proline for up to 72 h. Aliquots of the culture medium were withdrawn, and the proteins were precipitated with 10% perchloric acid. The supernatant was neutralized and treated with L-FDVA to derivatize the amino acids. The concentrations of *trans*-4-hydroxy-l-proline and *cis*-4-hydroxy-l-proline were determined by RP-HPLC–UV–MS, whereas the consumption of 4-oxo-l-proline was followed spectrophotometrically.

**Figure 10:**
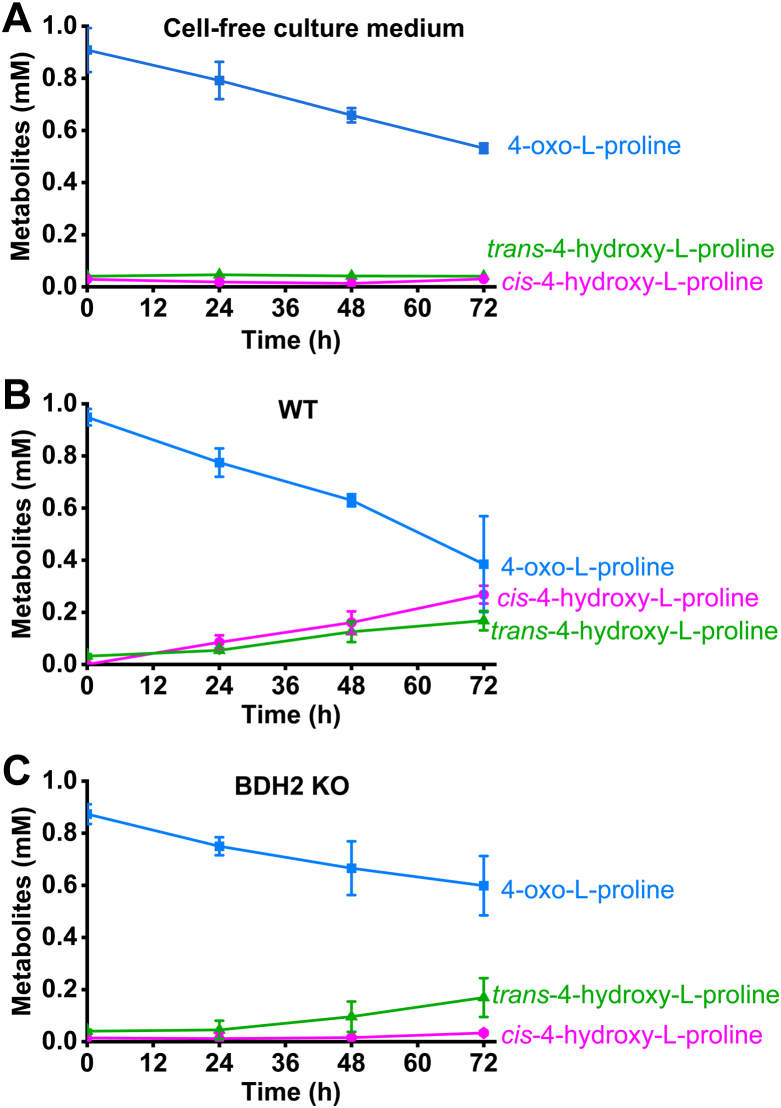
**Impact of BDH2 inactivation on the metabolism of 4-oxo-****l****-proline in HEK293T cells.** Changes in the extracellular concentration of 4-oxo-l-proline and its metabolites were measured in (*A*) a cell-free culture medium (cell-free medium), (*B*) WT, and (*C*) *Bdh2*-deficient HEK293T cells (*Bdh2*-KO), following supplementation of the medium with 1 mM 4-oxo-l-proline. The cells were plated in 6-well dishes and grown for 24 h, and this step was omitted in the cell-free variant of the experiment. After that time, the cell culture medium was supplemented with the oxoamino acid, and incubation was continued for up to 72 h, as described under “Experimental procedures” section. Values are the means ± SD (error bars) of three independent experiments performed with cells from three different culture passages or three different KO clonal cell lines (A12, A15, and B9, *cf.*Fig. 9) (n = 3). When no error bar is shown, the error is smaller than the width of the line. BDH2, type 2 (*R*)-β-hydroxybutyrate dehydrogenase; HEK293T, human embryonic kidney 293T cell line.

As shown in Figure 10, 4-oxo-l-proline was chemically unstable and gradually decomposed when incubated in the cell-free culture medium in the conditions used for culturing the cells over 72 h. The oxoamino acid concentration decreased by ≈0.4 to 0.5 mM after 72 h of incubation, but this decomposition did not coincide with the formation of *cis*-4-hydroxy-l-proline or *trans*-4-hydroxy-l-proline.

To gain information on the chemical nature of the spontaneous decomposition product, a larger scale (10 mM) of 4-oxo-l-proline was incubated in the cell-free culture medium, Hepes buffer (pH 7.4), or NH_4_HCO_3_ (pH 7.8) under the conditions used for cell culture for 72 h. Regardless of the incubation variant, each reaction solution gradually darkened, indicating the formation of a visible light–absorbing material. The UV–visible spectra of the product formed in NH_4_HCO_3_ showed absorption bands at 260 and 312 nm (Fig. S5), suggesting the formation of a new compound(s) with additional double bonds. Since the dark product immediately precipitated under acidic conditions, excluding the use of ESI–Q-TOF in positive ion mode for its analysis, the intact product formed in NH_4_HCO_3_ was further analyzed by RP–HPLC–photodiode array–Q-TOF in negative ion mode. This analysis revealed the formation of several compounds that differed in both chromatographic (Fig. S6) and spectral properties (Fig. S7). However, they exhibited poor negative-ion ESI, preventing their detailed characterization with Q-TOF MS (Fig. S8). These results suggest that 4-oxo-l-proline is a labile compound that most likely reacts with itself, leading to the formation of various chemical products. This conclusion consisted of previously reported data (18) showing that 4-oxo-l-proline undergoes spontaneous base-catalyzed aldol condensation, while its reactivity is the main obstacle in synthesizing its chemical derivatives.

The HEK293T cells reciprocally took up 4-oxo-l-proline from the culture medium and converted it into *cis*-4-hydroxy-l-proline found in the extracellular milieu (*cf.* Fig. 10). Formation of neither *cis*-4-hydroxy-l-proline nor *trans*-4-hydroxy-l-proline was detected in the absence of 4-oxo-l-proline (not shown). After 72 h of incubation, the extracellular concentration of 4-oxo-l-proline dropped by ≈0.6 mM, whereas *cis*-4-hydroxy-l-proline accumulated up to ≈0.3 mM. More intriguingly, the formation of the *cis*-isomer was accompanied by an accumulation of the *trans*-diastereomer at up to ≈0.2 mM concentration. A hypothetical conversion of *cis*-4-hydroxy-l-proline into the *trans*-isomer was unlikely based on the lack of evidence of this catalysis by mammalian enzymes. Thus, the observed consumption of 4-oxo-l-proline (≈0.6 mM) plausibly reflected the sum of its spontaneous chemical transformation and enzymatic conversion toward *cis*-4-hydroxy-l-proline. However, an alternative mechanism for the accumulation of *trans*-4-hydroxy-l-proline could be that 4-oxo-l-proline or products of its spontaneous chemical transformation could have exerted an inhibitory action on the intracellular breakdown of endogenous *trans*-4-hydroxy-l-proline, leading to its accumulation in the culture medium.

To verify these hypotheses, we investigated the metabolic fate of the *cis*-isomer in HEK293T cells. As shown in Fig. S9, the cells did neither convert *cis*-4-hydroxy-l-proline into *trans*-4-hydroxy-l-proline nor accumulate the latter metabolite in the culture medium, indicating both a lack of *cis*–*trans* isomerization of 4-hydroxy-l-proline and any impact of the *cis*-isomer on the metabolism of the endogenous *trans*-4-hydroxy-l-proline.

To confirm further that BDH2 is the sole enzyme responsible for converting 4-oxo-l-proline into *cis*-4-hydroxy-l-proline, we investigated the metabolism of the former compound in *Bdh2*-deficient HEK293T cells generated by the CRISPR–Cas9 method. The KO cells could not form *cis*-4-hydroxy-l-proline, as expected, whereas the accumulation of *trans*-4-hydroxy-l-proline was similar to that in the WT HEK293T cells (*cf.* Fig. 10).

Taken together, these results indicate that the BDH2 enzyme operates in the intact HEK293T cells, yielding *cis*-4-hydroxy-l-proline, a nonphysiological metabolite in mammals, whereas 4-oxo-l-proline or products of its spontaneous chemical transformation appear to inhibit the breakdown of endogenous *trans*-4-hydroxy-l-proline in HEK293T cells. However, we cannot completely exclude the presence of yet another enzymatic activity that specifically converts 4-oxo-l-proline to *trans*-4-hydroxy-l-proline in HEK293T cells.

### 4-Oxo-l-proline exerts a cytotoxic effect on HEK293T cells

A routine daily inspection of HEK293T cultures indicated that the cells incubated in the presence of 4-oxo-l-proline might show a higher death rate than the control ones. To test this possibility, the viability of the cells in the presence of 4-oxo-l-proline or *cis*-4-hydroxy-l-proline at 1 mM concentration was determined using the 3-[4,5-dimethylthiazol-2-yl]-2,5 diphenyl tetrazolium bromide (MTT) assay.

**Figure 11:**
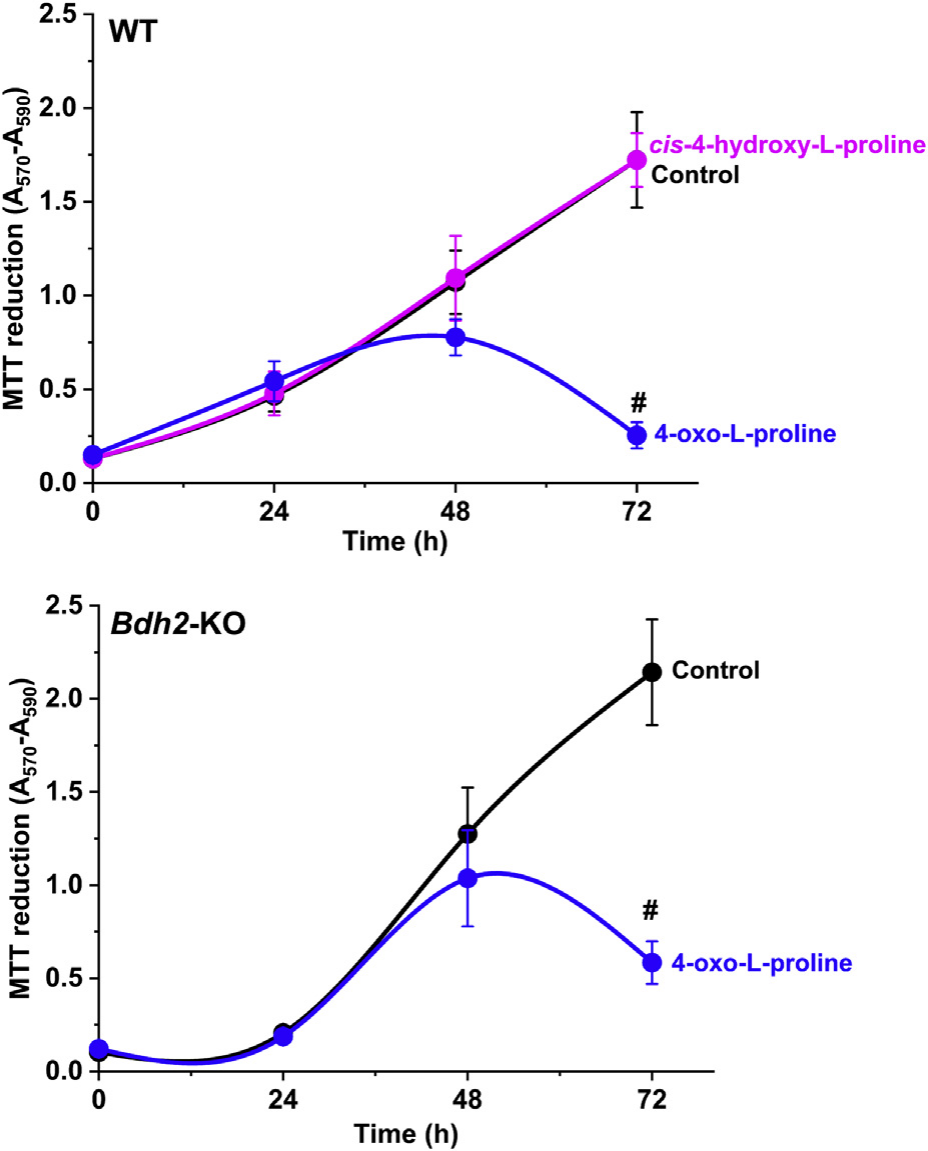
**The cell viability of HEK293T cells in the presence of 4-oxo-****l****-proline or *cis*-4-hydroxy-****l****-proline.** Cell viability was tested in WT and *Bdh2*-deficient HEK293T cells (*Bdh2*-KO) at the indicated time points by the MTT assay. The cells were seeded in 12-well dishes and grown for 24 h. After that time, the cell culture medium was replaced with the fresh one supplemented with 1 mM 4-oxo-l-proline or 0.3 mM *cis*-4-hydroxy-l-proline as indicated, and the incubation was continued for up to 72 h as described under “Experimental procedures” section. Values are the means ± SD (error bars) of three independent experiments performed in triplicates with cells from three different culture passages or three different KO clonal cell lines (A12, A15, and B9, *cf.*Fig. 9) (n = 3). The data were assumed to be distributed normally. Statistical significance was analyzed using a one-tailed paired Student's *t* test. ∗*p* < 0.02 and #*p* < 0.002 *versus* corresponding control values. HEK293T, human embryonic kidney 293T cell line; MTT, 3-[4,5-dimethylthiazol-2-yl]-2,5 diphenyl tetrazolium bromide.

Prolonged incubation of WT HEK293T cells with 4-oxo-l-proline led to a decrease in their viability by ≈70% compared with that for the control cells (Fig. 11). Surprisingly, incubation of the cells with *cis*-4-hydroxy-l-proline, an antiproliferative and a cytotoxic compound (12, 13), did not affect their viability, suggesting that the cytotoxic effect of 4-oxo-l-proline may be due to the oxoamino acid itself or its decomposition product(s) and not because of its conversion to *cis*-4-oxo-l-proline. To verify this possibility, we investigated the impact of 4-oxo-l-proline on the viability of *Bdh2*-deficient HEK293T cells that were unable to produce the *cis*-isomer (*cf.* Fig. 10). As shown in Figure 11, 4-oxo-l-proline decreased the viability of *Bdh2*-deficient HEK293T cells to a slightly larger extent (by ≈85%) than in the case of the WT cells, confirming further the cytotoxic activity of 4-oxo-l-proline or the products of its spontaneous chemical transformation in HEK293T cells.

## Discussion

Although the reaction catalyzed by 4-oxo-l-proline reductase is currently considered a normal part of metabolic pathways for l-proline degradation in mammals, as depicted in the Kyoto Encyclopedia of Genes and Genomes, for example (19), the identity of this enzyme and its biological importance are still unknown. Here, we report the identification of rat 4-oxo-l-proline reductase as BDH2, disclosing the identity of the mammalian enzyme. This conclusion is based on the following findings: (i) a multistep purification of the 4-oxo-l-proline reductase from rat kidneys resulted in the identification of a protein BDH2 as the only meaningful candidate for the enzyme; (ii) the recombinant rBDH2 and hBDH2 catalyzed the NADH-dependent reduction of 4-oxo-l-proline, yielding *cis*-4-hydroxy-l-proline *in vitro*. Mutants of these enzymes harboring mutations of the key catalytic residues are inactive, and (iii) the identity of the product made by the recombinant enzymes was confirmed by both RP chromatography and tandem MS. The identification of 4-oxo-l-proline reductase as BDH2 is also consistent with the findings that HEK293T cells endogenously expressing BDH2 protein efficaciously convert 4-oxo-l-proline to *cis*-4-hydroxy-l-proline, whereas *Bdh2*-deficient cells cannot metabolize the former to the latter. Importantly, no other enzyme has been shown to generate the *cis*-isomer of 4-hydroxy-l-proline in vertebrates yet (for review, see Ref. (10)).

BDH2 has a typical NAD(H)-binding Rossmann-fold domain in its structure and belongs to the short-chain dehydrogenase/reductase superfamily (Protein Data Bank code: 2AG5) (15). In humans, enzymes of this cluster are involved in the metabolism of many compounds, including steroid hormones, lipids, and xenobiotics (20). BDH2 was initially reported as a putative cytosolic BDH contributing to the oxidation of the ketone body β-hydroxybutyrate (15). However, the enzyme was reported to be very poor in oxidizing (*R*)-β-hydroxybutyrate, with a *K*_*M*_ of 12 mM and *k*_cat_ value of 1 min^−1^ at 50 mM concentration of the substrate (15). Also, these findings were confirmed in the present work. Such a low turnover number of the enzyme resembles that of an enzyme catalyzing post-translational modification rather than a metabolic one (21). These results strongly suggest that (*R*)-β-hydroxybutyrate cannot be a physiological substrate of BDH2. Indeed, no disturbances in the metabolism of ketone bodies were found in BDH2-deficient mice (22). Alternatively, BDH2 was proposed to be the homolog of bacterial EntA protein that oxidizes 2,3-dihydro-2,3-dihydroxybenzoic acid (2,3-diDHBA) to 2,3-dihydroxybenzoic acid and catalyzes the synthesis of 2,5-DHBA (gentisic acid), a putative mammalian siderophore (16). Intriguingly, BDH2 has never been shown in a direct experiment (*i.e.*, *in vitro*) to catalyze the production of 2,5-DHBA. Instead of this, the formation of 2,3-dihydroxybenzoic acid from 2,3-diDHBA in the presence of the enzyme was evidenced (16). It is also unclear which metabolic pathway could provide a putative substrate for the BDH2-dependent formation of 2,5-DHBA in mammalians. For these and other reasons, the physiological role of 2,5-DHBA and the importance of BDH2 for its synthesis were questioned experimentally by others (23).

We show in the present work that BDH2 catalyzes the reversible reduction of 4-oxo-l-proline to *cis*-4-hydroxy-l-proline, with a high substrate specificity and catalytic activity. The formation of *cis*-4-hydroxy-l-proline is remarkable as no enzyme has been shown to catalyze the production of the *cis*-isomer in vertebrate species to date. It is also evident now that the irreversibility of this reaction reported by Smith and Mitoma (5) was due to the use of *trans*-4-hydroxy-l-proline instead of its *cis*-isomer in enzymatic tests. Concerning the plausible substrates, neither 5-oxo-l-proline nor *trans*-4-hydroxy-l-proline was accepted, indicating a preferential requirement for the presence of a *cis*-OH or keto group at carbon 4. Furthermore, the catalytic efficiency (*k*_cat_/*K*_*M*_) of the human enzyme on 4-oxo-l-proline (2450 min^−1^ × mM^−1^) was 12,500-fold higher than that on (*R*)-β-hydroxybutyrate (0.195 min^−1^ × mM^−1^), confirming that the latter compound is unlikely to be a physiological substrate. In contrast, a negligible activity toward (*R*)-β-hydroxybutyrate and l-threonine and no activity on acetoacetate imply that cyclic compounds are much better substrates for BDH2 than the linear ones. Unfortunately, no information on the specific activity and the kinetic parameters of BDH2 for 2,3-diDHBA was reported by Devireddy *et al.* (16). Thus, we could not compare the catalytic activity of the enzyme on 2,3-diDHBA to that for 4-oxo-l-proline.

**Figure 12:**
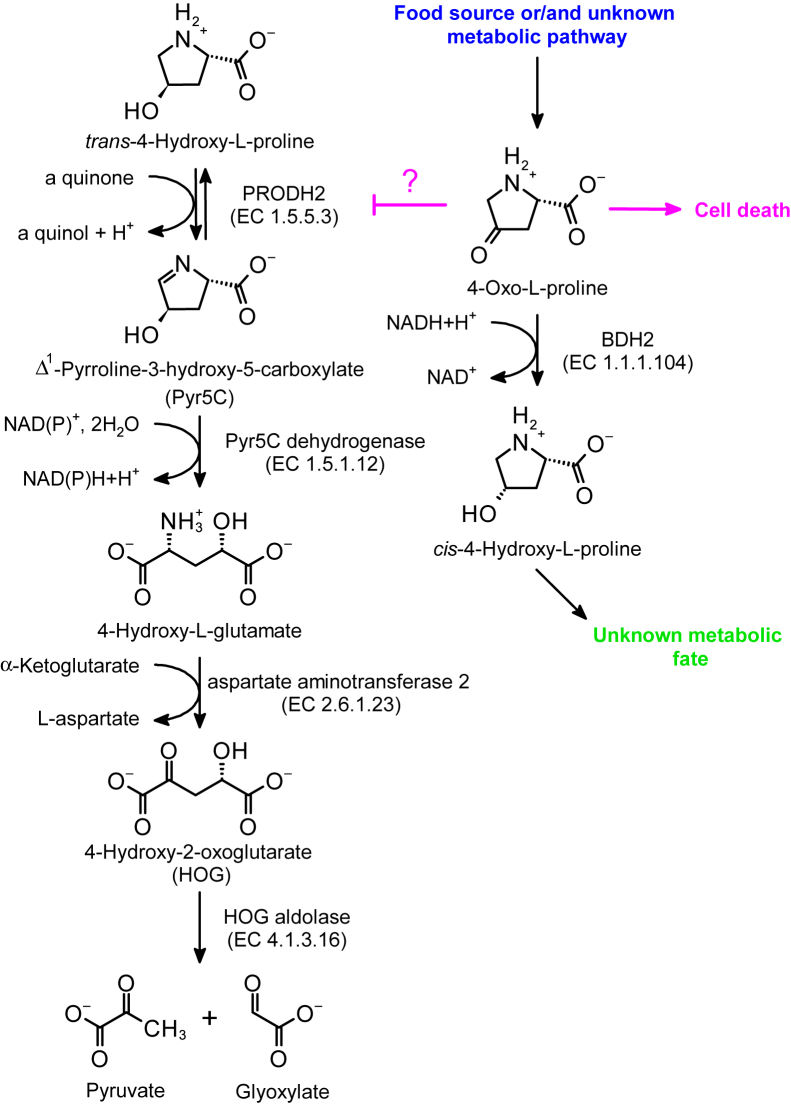
**The hypothesized relationship between metabolism of 4-oxo-****l****-proline and *trans*-4-hydroxy-****l****-proline in HEK293T cells.** 4-Oxo-l-proline decreases cell viability by a yet unknown mechanism and inhibits degradation of endogenous *trans*-4-hydroxy-l-proline, most likely by decreasing the activity of proline dehydrogenase 2 (PRODH2). BDH2 catalyzes the reduction of 4-oxo-l-proline to *cis*-4-hydroxy-l-proline, thereby eliminating the cytotoxic metabolite. BDH2, type 2 (*R*)-β-hydroxybutyrate dehydrogenase; HEK293T, human embryonic kidney 293T cell line.

The results presented here identified 4-oxo-l-proline as a novel substrate for BDH2. Although this oxoamino acid is a poorly studied metabolite, it is occasionally detected in metabolomics experiments as a constituent of human plasma (3, 24) and cells (2, 25). The origin of 4-oxo-l-proline is currently unclear, but the finding that its intracellular concentration doubled in human T lymphocytes incubated with a high concentration of l-arginine suggests that 4-oxo-l-proline is indeed an endogenous metabolite, most likely contributing to l-arginine/l-proline metabolic pathways (25). Also, 4-oxo-l-proline might come from food as it is detected in dairy products (26). An alternative explanation would be that 4-oxo-l-proline is endogenously produced from *cis*-4-hydroxy-l-proline in the reverse reaction catalyzed by BDH2 (Fig. 12). This enzyme is undoubtedly capable of catalyzing such a reaction *in vitro* in the presence of NAD^+^ at a 1 mM concentration that is well within the physiological values reported in human cells (0.2–1 mM, (27)). However, it should be noted that no conversion of *cis*-4-hydroxy-l-proline into 4-oxo-l-proline was detected in HEK293T cells incubated with the former compound, suggesting that the reduction of 4-oxo-l-proline is the preferred reaction *in vivo* under the conditions used in this study.

We show in the present work that the intracellular conversion of 4-oxo-l-proline to *cis*-4-hydroxy-l-proline in HEK293T cells solely depends on the activity of BDH2. In addition, this enzyme is certainly not responsible for the accumulation of *trans*-4-hydroxy-l-proline when the cells were incubated with 4-oxo-l-proline. This is directly evidenced by the finding that the loss of BDH2 protein in the KO cells has no impact on the production of *trans*-4-hydroxy-l-proline. Thus, although our data do not completely exclude the possibility that *trans*-4-hydroxy-l-proline comes directly from 4-oxo-l-proline because of a novel enzymatic activity in HEK293T cells, we are postulating instead that 4-oxo-l-proline or a product of its spontaneous chemical transformation might act by blocking the degradation pathway of *trans*-4-hydroxy-l-proline in HEK293T cells, resulting in its accumulation in the culture medium. Although the results do not allow us to identify the inhibited enzyme(s), it is not unwise to hypothesize that 4-oxo-l-proline might be an inhibitor of hydroxyproline dehydrogenase (proline dehydrogenase 2 [PRODH2]) that converts *trans*-4-hydroxy-l-proline to Δ^1^-pyrroline-3-hydroxy-5-carboxylate (Pyr5C), hence initiating the degradation of the endogenous *trans*-4-hydroxy-l-proline in mitochondria (*cf.* Fig. 12). The reasoning behind this notion is as follows: (i) 4-oxo-l-proline is a very close structural analog of only two metabolites in the *trans*-4-hydroxy-l-proline and l-proline catabolic pathways: *trans*-4-hydroxy-l-proline and Pyr5C, hence it is likely to inhibit either PRODH2 or Pyr5C dehydrogenase, (ii) PRODH2 is an enzyme unique to the hydroxy-amino acid breakdown pathway, whereas Pyr5C dehydrogenase is shared between the *trans*-4-hydroxy-l-proline and l-proline catabolic pathways (26, 27), and (iii) 4-oxo-l-proline did not exert any effect on the concentration of l-proline in the cell culture medium derived from the WT and *Bdh2*-deficient HEK293T (data not shown), indicating that 4-oxo-l-proline or its spontaneous decomposition product(s) most likely affect an enzyme related specifically to the degradation of *trans*-4-hydroxy-l-proline, that is, PRODH2.

Most importantly, the aforedescribed arguments are in perfect agreement with findings of an early study on 4-oxo-l-proline metabolism in animals by Mitoma *et al.* (4). They found that 4-oxo-l-proline inhibited the breakdown of 4-hydroxy-l-proline and did not affect the catabolism of l-proline in isolated mammalian mitochondria.

Furthermore, that inhibition would resemble the biochemical phenotype associated with the deficiency of hydroxyproline dehydrogenase, a benign metabolic disorder (28). Interestingly, analogs of *trans*-4-hydroxy-l-proline were tested as potential inhibitors of PRODH2, including *trans*-4-hydroxy-d-proline, *cis*-4-hydroxy-l-proline, and *cis*-4-hydroxy-d-proline. However, none of them inhibited the reaction at concentrations >5 mM (29). Further studies are thus needed to evaluate the inhibitory effect of 4-oxo-l-proline and its analogs on PRODH2 activity as this enzyme could have therapeutic value in primary hyperoxalurias (29).

Our data show that 4-oxo-l-proline exerts a potent cytotoxic effect on HEK293T cells, though the mechanism and exact metabolite responsible for this toxicity remains unknown. Although we are not able to conclude now whether this effect is due to 4-oxo-l-proline itself or the action of the product(s) of its chemical decomposition, it is tempting to speculate that the physiological role of BDH2 might be to eliminate 4-oxo-l-proline, thereby preventing its toxicity and interference with the catabolic pathway of *trans*-4-hydroxy-l-proline.

In conclusion, we have shown here that mammalian BDH2 is 4-oxo-l-proline reductase that catalyzes the reversible reduction of 4-oxo-l-proline to *cis*-4-hydroxy-l-proline and not *trans*-4-hydroxy-l-proline as currently believed. BDH2 is, therefore, the first enzyme capable of producing the *cis*-isomer of 4-hydroxy-l-proline to be identified in vertebrates. BDH2 also allows intact HEK293T cells to metabolize 4-oxo-l-proline to *cis*-4-hydroxy-l-proline. Since 4-oxo-l-proline and/or the product(s) of its spontaneous chemical transformation are cytotoxic, this reaction pathway might be considered for eliminating the toxic metabolite(s). Finally, this work also shows that 4-oxo-l-proline or its spontaneous decomposition product(s) might interfere with the metabolism of the endogenous *trans*-4-hydroxy-l-proline in human cells.

## Experimental procedures

### Materials

Reagents of analytical grade, whenever possible, were from Sigma or Merck. 4-oxo-l-proline hydrobromide was purchased from Alfa Aesar (90+% purity) or Fluorochem (95% purity). Q-Sepharose FF resin, HiScreen Blue FF, Superdex 200 16/60 HiLoad, and HisTrap FF crude columns were obtained from GE Healthcare Bio-Sciences. Vivaspin 20 centrifugal concentrators were from Sartorius. Enzymes and DNA-modifying enzymes were obtained from Thermo-Fermentas, A&A Biotechnology, or Bio-Shop.

### Assay of 4-oxo-l-proline reductase activity

The enzyme activity was determined by the modified method employed previously (5). Briefly, the activity was followed spectrophotometrically at 37 °C by measuring the rate of NADH conversion into NAD^+^, which is accompanied by a decrease in absorbance at λ = 340 nm (ε = 6.22 mM^−1^ cm^−1^). The standard incubation mixture (1 ml) contained 80 mM Na^+^ phosphate, pH 6.5; 1 mM DTT; 0.2 mM NADH; and 2 mM 4-oxo-l-proline. The latter was prepared as a fresh 25 mM solution, and pH was adjusted to 6.0 with 1 M NaHCO_3_ and filtered using 0.22 μm Spin-X tube filters (cellulose acetate). The actual concentration of 4-oxo-l-proline in the stock solution was verified spectrophotometrically. The reaction was started by adding the enzyme preparation and carried out at 37 °C for 15 min unless otherwise described. Kinetic properties and substrate specificity of the BDH2 enzymes were determined in the standard incubation mixture supplemented with 0.1 mg/ml bovine serum albumin. Oxidase activity of both enzymes was determined in the analogical buffer with 50 mM Tris–HCl, pH 9.0, in the presence of 1 mM NAD^+^ instead of 0.2 mM NADH. All reactions were linear for at least 10 min under all studied conditions.

### Purification of rat 4-oxo-l-proline reductase

Eighteen male Wistar Albino Glaxo rats, aged 3 months, were purchased from the Animal House of the Mossakowski Medical Research Centre, Polish Academy of Sciences. The animals were euthanized by a carbon dioxide euthanasia (Directive 2010/63/European Union of the European Parliament). Rat kidneys (40 g) were homogenized in a Waring Blender 7011HS (4 cycles × 30 s with a pause of 10 s) with three volumes (w/v) of a buffer consisting of 20 mM Tris–HCl, pH 8.0, 1 mM DTT, 20 mM KCl, and 4 μg/ml leupeptin. The homogenate was centrifuged for 20 min at 15,000*g* at 4 °C. The resulting supernatant (120 ml) was then fractionated between 0% and 10% concentration (w/v) of polyethylene glycol 4000. After 15 min incubation on ice, the sample was centrifuged for 10 min at 15,000*g* at 4 °C. The supernatant was again submitted for fractionation with PEG-4000 concentration (w/v) between 10% and 25%. After 15 min incubation on ice, the sample was centrifuged for 10 min at 15,000*g* at 4 °C. The 10 to 25% precipitate was dissolved in 120 ml of homogenization buffer and frozen at −70 °C before purification.

Clarified sample 10 to 25% was applied to a Q Sepharose column (100 ml) equilibrated with buffer A consisting of 20 mM Tris–HCl, pH 8.0, 1 mM DTT, 20 mM KCl, and 1 μg/ml leupeptin. The column was washed with 240 ml of buffer A, developed with a linear NaCl gradient (0–1 M in 400 ml) in buffer A, and fractions (5 ml) were collected. The most active fractions from the Q Sepharose column were pooled (20 ml), diluted to 54 ml with buffer C (80 mM Na^+^ phosphate, pH 6.5, 1 mM DTT, 20 mM KCl, and 0.5 mM PMSF), and applied to a HiScreen Blue FF column (4.7 ml) equilibrated with buffer C. The column was washed with 56 ml of buffer C, developed with a linear NaCl gradient (0–1.5 M in 51 ml) in buffer C, and fractions (3 ml) were collected. The most active fractions from the HiScreen Blue FF column (12 ml) were pooled, concentrated to 4.5 ml using Vivaspin 20 ultrafiltration unit, and 2 ml of the sample was loaded on a Superdex 200 16/600 HiLoad column (120 ml) equilibrated with buffer D consisting of 80 mM Na^+^ phosphate, pH 6.5, 1 mM DTT, 20 mM KCl, and 0.1 M NaCl. The gel filtration column was then developed with 140 ml of buffer C, and 2 ml fractions were collected. All purification steps were performed at 4 °C, and the enzymatic preparation was stored at −70 °C between steps.

### Identification of the rat 4-oxo-l-proline reductase by tandem MS

The protein bands of the most active fractions coeluting with 4-oxo-l-proline reductase activity in the Superdex 200 16/600 HiLoad purification step were cut from a 10% polyacrylamide SDS gel and digested with trypsin. Appropriate negative controls from two fractions lacking the activity were prepared as well. In-gel digestions of the peptides were performed as described previously (30). Peptides were analyzed by nanoUPLC-tandem MS employing Acquity nanoUPLC coupled with a Synapt G2 HDMS Q-TOF mass spectrometer (Waters) fitted with a nanospray source and working in MSˆE mode under default parameters. Briefly, the products of in-gel protein digestion were loaded onto a Waters Symmetry C18 trapping column (20 mm × 180 μm) coupled to the Waters BEH130 C18 UPLC column (250 mm × 75 μm). The peptides were eluted from columns in a 1 to 85% gradient of acetonitrile in water (both containing 0.1% formic acid) at a flow rate of 0.3 μl/min. The peptides were directly eluted into the mass spectrometer. Data were acquired and analyzed using MassLynx 4.1 software (Waters) and ProteinLynx Global Server 2.4 software (PLGS; Waters) with a false positive rate of ≤4% as implemented by PLGS software.

The raw data were processed employing Apex3D algorithm implemented in PLGS software with the following settings: (i) chromatographic peak width and MS TOF resolution: automatic, (ii) lock mass window: 0.25 Da, (iii) the low and elevated energy thresholds: 250 and 100 counts, respectively, and (iv) the intensity threshold: 1500 counts.

For peptide and protein identification, an MSˆE search was performed against a randomized database specified later. The peptide and fragment mass tolerance was set to automatic mode. The minimum number of fragment ion matches required for a peptide was three; the minimum number of fragment ion matches required for a protein was 7, and the minimum number of peptide matches required for a protein to remain under consideration was set to 1. The primary digest reagent was trypsin, and at most, one missed cleavage was permitted. No secondary reagent was specified. About 200 kDa was set as the maximum protein mass under consideration. The carbamidomethylation of cysteine was the only specified fixed modification, whereas the oxidation of methionine was applied as the variable modification.

To identify 4-oxo-l-proline reductase, the complete rat (*Rattus norvegicus*) reference proteome (66,853 entries) was downloaded on July 24, 2018, from the NCBI Protein database, randomized, and used as a databank for the MS/MS software.

### Overexpression and purification of the recombinant BDH2 proteins and inactive mutants

Rat total RNA was prepared from 100 mg of kidneys using TriPure reagent (Roche) according to the manufacturer's instructions. Complementary DNA was synthesized using Moloney murine leukemia virus reverse transcriptase (TranScriba, A&A Biotechnology), with oligo(dT)_18_ primer and 2.5 mg total RNA according to the manufacturer's instructions. The ORF-encoding human enzyme was purchased from DNASU Plasmid Repository (cloneID: HsCD00640151). The ORFs encoding rat (NCBI Reference Sequence: NM_001106473.1) and human (NM_020139.4) BDH2 protein were PCR amplified using Pfu DNA polymerase. Both rat and human ORFs coding for BDH2 were amplified using specific 5′ primers containing the initiator codon included in the NdeI site and 3′ primers containing the stop codon flanked by a KpnI site (for primer sequence, refer to Table 5).

**Table 5:** Sequences of primers used for PCR amplification of BDH2 ORFs and site-directed mutagenesis experiments

Primer	Sequence	Restriction site	Protein expressed
WT BDH2 proteins			
rBDH2-pCOLD-S	tatacatATGTTTTTGGATTGCACTGCAGG	NdeI	*N*-terminal His_6_-tagged rBDH2
rBDH2-pCOLD-AS	tataggtaccTCACAGACTCCAACCGCCATC	KpnI	
hBDH2-pCOLD-S	taatcatATGGGTCGACTTGATGGGAAAG	NdeI	*N*-terminal His_6_-tagged hBDH2
hBDH2-pCOLD-AS	taaggtaccTCACAAGCTCCAGCCTCCATC	KpnI	
Site-directed mutagenesis of BDH2 proteins			
Y157F-rBDH2-S	GAGAACAGATGTGTG**TTC**AGTGCAACCAAGG	N/A	*N*-terminal His_6_-tagged mutated form of rBDH2
Y157F-rBDH2-AS	CCTTGGTTGCACT**GAA**CACACATCTGTTCTC	N/A	
Y147F-hBDH2-S	CAGATGTGTG**TTC**AGCACAACCAAGGCAGC	N/A	*N*-terminal His_6_-tagged mutated form of hBDH2
Y147F-hBDH2-AS	GCTGCCTTGGTTGTGCT**GAA**CACACATCTG	N/A	

The nucleotides corresponding to the coding sequences are in capital letters, restriction sites are underlined, and mutated codons are shown in boldface type.

Abbreviation: N/A, not applicable.

The amplified DNA products of the expected size were digested with the appropriate restriction enzymes and cloned into the pCOLD I expression vector (Takara Bio), which allows for the production of proteins with an *N*-terminal His_6_ tag. All constructs were verified by DNA sequencing.

For protein production, *Escherichia coli* BL21(DE3) cells were transformed with an appropriate DNA construct, and a single colony was selected to start an overnight preculture. About 300 ml of LB broth (with 100 mg/ml ampicillin) was inoculated with 30 ml of the preculture and incubated (37 °C, 175 rpm) until an absorbance of 0.5 at 600 nm was reached. The culture was placed on ice for 20 min (cold shock) to induce protein expression. Next, cells were incubated for 16 h at 13 °C, 175 rpm, and harvested by centrifugation (6000*g* for 10 min). The cell paste was suspended in 15 ml lysis buffer consisting of 80 mM Na^+^ phosphate, pH 6.5, 1 mM DTT, 20 mM KCl, 1 mM PMSF, 5 μg/ml leupeptin, 5 μg/ml antipain, 0.25 mg/ml hen egg-white lysozyme (BioShop), and 250 U of Viscolase (A&A Biotechnology). The cells were lysed by freezing in liquid nitrogen and, after thawing and vortexing, the extracts were centrifuged at 4 °C (20,000*g* for 20 min).

For the purification of recombinant BDH2 proteins, the supernatant of *E. coli* lysate (15 ml) was diluted threefold with buffer A (100 mM Na^+^ Hepes [pH 8.0], 200 mM NaCl, 30 mM imidazole, 1 μg/ml leupeptin, and 1 μg/ml antipain) and applied onto a HisTrap FF crude column (1 ml) equilibrated with the same buffer. The column was then washed with 10 ml buffer A, and the retained proteins were eluted with a stepwise gradient of imidazole (5 ml of 90 mM, 5 ml of 180 mM, and 5 ml of 300 mM) in buffer A. The recombinant proteins were present in both 180 and 300 mM imidazole fractions. Both fractions exhibited >95% purity, as confirmed by SDS-PAGE. The enzyme preparation was submitted to dialysis against buffer consisted of 80 mM Na^+^ phosphate, pH 7.5, 1 mM DTT, 100 mM NaCl, 6% sucrose, 1 μg/ml leupeptin, and 1 μg/ml antipain. The purified proteins were aliquoted and stored at −70 °C.

Mutated forms of rBDH2 and hBDH2 enzymes (Y157F and Y147F, respectively) were generated by site-directed mutagenesis using a QuikChange II XL kit (Stratagene), with pCOLD I/rBDH2 and pCOLD I/hBDH2 plasmids as templates and mutagenic primers Y157F-rBDH2-S and Y157F-rBDH2-AS as well as Y147F-hBDH2-S and Y147F-hBDH2-AS, respectively (Table 5). Both mutated forms of BDH2 were then produced in *E. coli* BL21(DE3) and purified using HisTrap FF crude column (1 ml) as described for the WT enzymes (data not shown).

### Generation of the HEK293T cell lines deficient in BDH2 by CRISPR–Cas9 method

The HEK293T cell is a hypotriploid cell line, with the modal chromosome number equal to 64. The copy number of chromosome 4, where the *Bdh2* gene is located, may vary between cells, and monosomy or a complete loss of chromosome 4 is frequently detected in some cells (31). Since the clonal line of HEK293T cell (from European Collection of Authenticated Cell Cultures *via* Sigma–Aldrich) cultured in our laboratory efficiently expressed the BDH2 protein, as confirmed by Western blotting (*cf.* Fig. 9), this parental cell line was used to create human cells deficient in BDH2 by CRISPR–Cas9 technology.

The CRISPR–Cas9 construct was generated to target exon 4 of the hBDH2 gene by ligating a pair of annealed primes (hBDH2_CRISPR_S: caccgGAAGGAATGCCTTGATCATC and hBDH2_CRISPR_AS: aaacGATGATCAAGGCATTCCTTCc) into the vector eSpCas9(1.1)-T2A-Puro digested by BbsI. This vector encodes an enhanced specificity Cas9 enzyme (a gift from Andrea Németh; Addgene; plasmid no.: 101039) (32). The construct was validated by sequencing (Genomed). HEK293T cells were cultured in Dulbecco's minimal essential medium (DMEM) supplemented with 100 units/ml penicillin, 100 g/ml streptomycin, and 10% (v/v) fetal bovine serum, and grown in a humidified incubator under a 95% air and 5% CO_2_ atmosphere at 37 °C. Cells were transfected with the CRISPR construct precisely as described (33). Genomic DNA from puromycin-resistant clones was used to PCR amplify the regions encompassing the targeted site, and the PCR products were sequenced to evaluate the gene modification present in each clone. The three BDH2-KO clones that were ultimately selected for further studies presented different 3-bp deletions within the genomic sequence coding for Leu111-Met112-Ile113, resulting in homozygous or heterozygous mutations and a specific loss of Met112. Surprisingly, no frameshifts or premature stop codons were detected. The analyses were performed employing the DSDecode tool for automatic decoding of superimposed chromatograms derived from PCR amplicons (34) and verified with the use of the Indigo tool from the GEAR Web site (https://www.gear-genomics.com) (35).

The absence of BDH2 protein in BDH2-KO clonal cell lines was confirmed by Western blotting performed as described previously (36) with the use of a polyclonal rabbit antibody against BDH2 (catalog no.: PA5-44760; Invitrogen) and a horseradish peroxidase–conjugated goat anti-rabbit secondary immunoglobulin G antibody (catalog no.: AS09602; Agrisera).

### Product analysis

#### Products of enzymatic reaction

To obtain products formed in the reactions catalyzed by recombinant BDH2 proteins for MS analysis, 2 μg of the homogenous recombinant enzyme was incubated in the reaction mixture (1 ml) containing 10 mM Na^+^ phosphate, pH 6.5; 1 mM DTT; 0.2 mM NADH, and 2 mM 4-oxo-l-proline. The reaction was started by the addition of BDH2 and followed up spectrophotometrically as described previously. After 0 and 15 min of incubation at 37 °C, 0.9 ml of the reaction mixture was withdrawn and transferred to 200 μl of ice-cold 10% (w/v) HClO_4_ to stop the reaction. Samples were centrifuged at 13,000*g* for 10 min at 4 °C, and the supernatants (1.1 ml) were immediately withdrawn and neutralized with 70 μl of 3 M KOH/3 M KHCO_3_. The salt precipitate was removed by centrifugation (13,000*g* for 10 min).

The clear supernatants were subjected to precolumn chiral derivatization of 4-hydroxy-l-proline isomers with L-FDVA, whereas RP-HPLC analysis accomplished the separation of derivatized amino acids according to a slightly modified method described by Langrock *et al.* (17). Briefly, 25 μl of neutralized supernatant was mixed with 10 μl of 1 M NaHCO_3_, followed by 40 μl of 35 mM L-FDVA (dissolved in acetone). The mixture was incubated for 90 min at 40 °C, and the reaction was stopped by adding 10 μl 1 M HCl. Next, the mixture was diluted with 50 μl acetonitrile and 115 μl water, and the samples were stored at −20 °C before further analysis. The amino acid derivatives were separated in a gradient mode on the Zorbax SB-C18 column (ODS; 4.6 × 250 mm, particle size of 5 μm) using Waters HPLC 600 system equipped with Waters 2487 UV detector and Waters Synapt G2 HDMS Q-TOF mass spectrometer fitted with an electrospray source. Mobile phases consisted of solvent A, containing 0.1% formic acid in the water, and solvent B, containing 0.1% formic acid in acetonitrile. The separation was performed in a linear gradient from 20 to 30% of solvent B for 30 min and subsequently, from 30 to 65% for 19 min at a flow rate of 1 ml/min, followed by the column equilibration for a further 11 min under the initial conditions. The UV detector monitored the column eluate at λ = 340 nm, followed by the mass spectrometer, operating in positive ESI–MS or ESI–MS/MS mode. The mass spectral data were recorded for *m/z* = 100 to 500 to detect L-FDVA and derivatives of l-proline and 4-hydroxyprolines. The ESI source was set at a temperature of 100 °C, the capillary voltage of 3.5 kV, and the cone voltage of 28 V. The flow rate of the desolvation gas (nitrogen) was 1100 l/h, and the desolvation temperature was 250 °C. To confirm the structure of the L-FDVA-derivatized *cis*-4-hydroxy-l-proline or *trans*-4-hydroxy-l-proline precursor ions, collision-induced dissociation experiments were run by selecting the target ion (*m/z* 412). The transfer collision energy ramp was set from 5.0 to 20.0 eV. Quantification was achieved using external standards of all studied amino acids after their derivatization with L-FDVA.

The aforedescribed method did not allow us to obtain an L-FDVA-derivatized 4-oxo-l-proline. This is plausibly because of the chemical instability of 4-oxo-l-proline in the alkaline condition required for derivatization reaction (5).

#### Products of 4-oxo-l-proline metabolism in mammalian cell cultures

To obtain products of 4-oxo-l-proline or *cis*-4-hydroxy-l-proline metabolism in HEK293T cells for MS analysis, the cells were plated in 6-well dishes (9.5 cm^2^) at a cell density of 0.60 × 10^6^ cells/well, respectively, in DMEM supplemented with 100 units/ml penicillin, 100 g/ml streptomycin, and 10% (v/v) fetal bovine serum, and grown in a humidified incubator under a 95% air and 5% CO_2_ atmosphere at 37 °C.

Twenty-four hours after seeding the cells, the culture medium was changed to a fresh one (2 ml) and supplemented with PBS (control), 1 mM 4-oxo-l-proline, or 0.3 mM *cis*-4-hydroxy-l-proline. Next, the cells were incubated for 0, 24, 48, or 72 h before transferring the medium (1 ml) to 100 μl of 35% (w/v) HClO_4_. Precipitated protein was separated by centrifugation (13,000*g* for 10 min). After neutralization of the supernatant with 3 M KOH/3 M KHCO_3_, the salts were removed by centrifugation (13,000*g* for 10 min); the clear supernatant was subjected for derivatization with L-FDVA to determine the products of 4-oxo-l-proline and *cis*-4-hydroxy-l-proline metabolism as described previously. Finally, the consumption of 4-oxo-l-proline was measured spectrophotometrically with the use of recombinant BDH2.

### MTT cell viability assay

HEK293T cells were seeded in 12-well dishes (3.8 cm^2^) at a cell density of 60 × 10^3^ in 1 ml DMEM supplemented with 100 units/ml penicillin, 100 g/ml streptomycin, and 10% (v/v) fetal bovine serum, and grown in a humidified incubator under a 95% air and 5% CO_2_ atmosphere at 37 °C.

Twenty-four hours after seeding the cells, the culture medium was changed to a fresh one (1 ml) and supplemented with either PBS (control), 1 mM 4-oxo-l-proline, or 1 mM *cis*-4-hydroxy-l-proline in PBS, and cultured further for 0, 24, 48, or 72 h. To determine the cell viability, 100 μl of MTT (5 mg/ml in PBS) was added to each well, and the cells were incubated for an additional 30 min in the CO_2_ incubator. After removing the culture medium, the intracellular crystals of MTT–formazan were completely solubilized in 1 ml of isopropanol:0.1 M HCl (90:10, v/v). The resulting purple solution was centrifuged (13,000*g* for 10 min) to remove cell debris, and its absorbance was measured spectrophotometrically at λ = 570 nm (formazan) and 690 nm (37).

### Analytical methods

Protein concentration was determined spectrophotometrically according to Bradford (38) using bovine γ-globulin as a standard. When appropriate, the His_6_-tagged recombinant proteins were detected by Western blot analysis, employing a mouse primary monoclonal antibody against His_6_-tag (catalog no.: MA1-4806; Invitrogen) and a horseradish peroxidase–conjugated goat antimouse antibody (catalog no.: A2554; Sigma–Aldrich), as described previously (21). All Western blotting analyses employed chemiluminescence and signals acquisition with Amersham Hyperfilm ECL, with the pattern of the prestained protein ladder being copied from the blotting membrane onto the film using a set of felt-tip pens.

### Calculations

*V*_max_, *K*_*M*_, and *k*_cat_ for reductase activities of the studied enzymes were calculated with Origin 2020 software (OriginLab) using nonlinear regression. All data are presented as mean ± SD.

## Data availability

The MS proteomics data are available at Zenodo repository (https://zenodo.org/) with the dataset identifier http://doi.org/10.5281/zenodo.5091801. The complete list of identified proteins and assigned peptides is shown in File S1. All other data are contained within the article.

## Supporting information

This article contains supporting information.

## Conflict of interest

The authors declare that they have no conflicts of interest with the contents of this article.
