# Supplementary material for: Recharacterization of the mammalian cytosolic type 2 (R)-β-hydroxybutyrate dehydrogenase as 4-oxo-l-proline reductase (EC 1.1.1.104)
Source: J Biol Chem. 2022 Feb 10;298(3):101708. doi: 10.1016/j.jbc.2022.101708 (PMC8914325; doi:10.1016/j.jbc.2022.101708)
Supplement: Supplemental Figures S1–S9 [file mmc1.pdf]

Recharacterization of the Mammalian Cytosolic Type 2 (*R*)-β-Hydroxybutyrate Dehydrogenase (BDH2) as 4-Oxo-L-Proline Reductase (EC 1.1.1.104)

Sebastian Kwiatkowski[1], Maria Bozko[1], Michal Zarod[1], Apolonia Witecka[1], Kubra Kocdemir[1], Adam K. Jagielski[1], and Jakub Drozak[1]*

*Corresponding author: Jakub Drozak

E-mail: jdrozak@biol.uw.edu.pl

List of supporting informations:

Figures S1 to S9

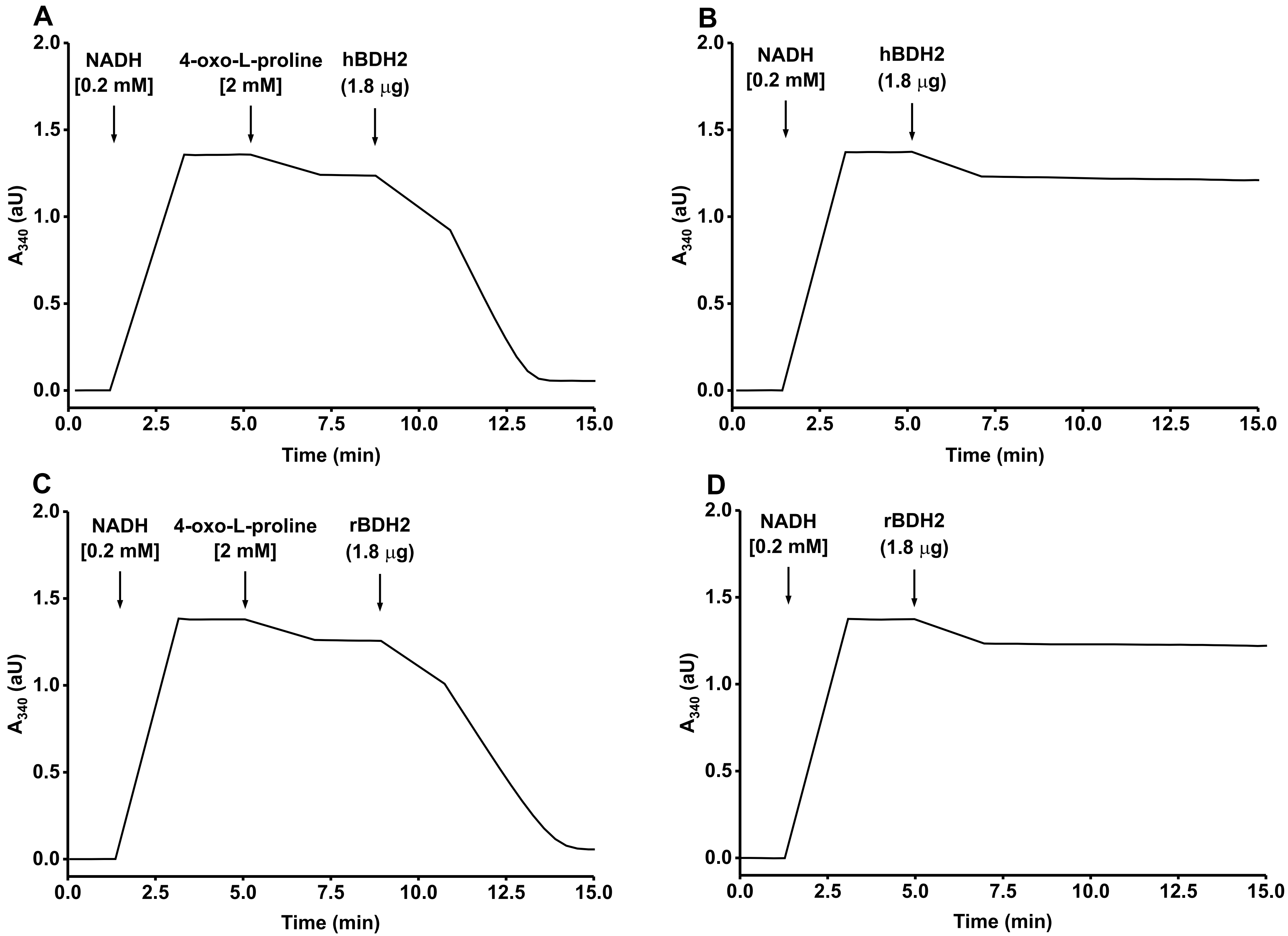

Fig. S1. Test of the purified recombinant BDH2 activity. The activity of human (hBDH2) and rat (rBDH2) enzymes was followed spectrophotometrically by measuring the conversion of NADH into NAD^+ (λ = 340 nm). The reactions were performed as described in the "*Experimental Procedures*" section. **A.** and **C.** The addition of 1.8 μg of the enzyme to the reaction mixture containing 4-oxo-L-proline resulted in the complete oxidation of the NADH as indicated by the change in the absorbance (A_{340}), **B.** and **D.** whereas that reaction was undetectable in the absence of the substrate.

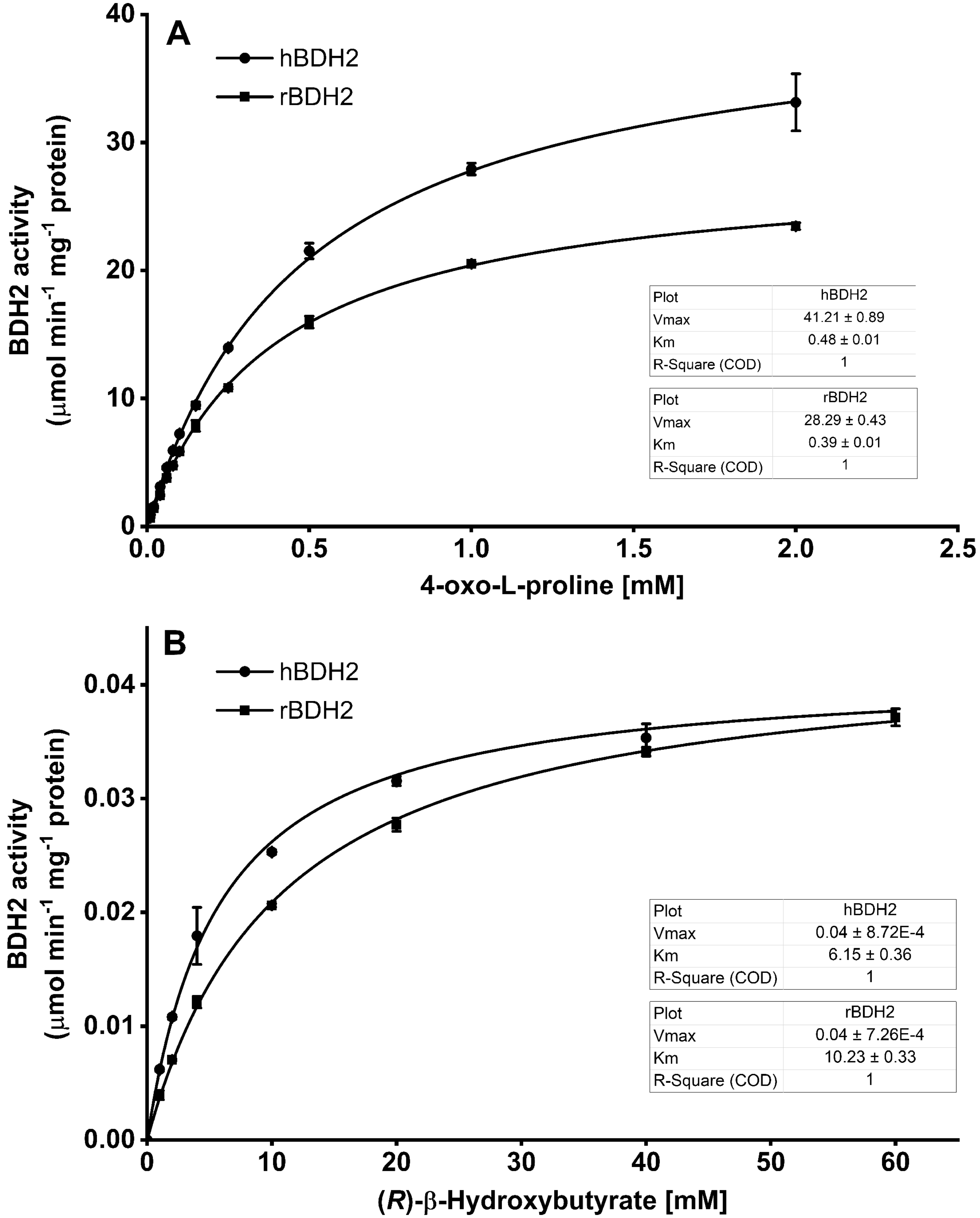

Fig. S2. Michaelis-Menten equation curves for the reactions catalyzed by human and rat BDH2. The activity of human (hBDH2) and rat (rBDH2) enzymes was followed spectrophotometrically by **A.** measuring the conversion of NADH into NAD^+ in the presence of 4-oxo-L-proline at 0.01, 0.02, 0.04, 0.06, 0.08, 0.1, 0.15, 0.25, 0.5, 1 and 2 mM concentration, or **B.** NAD^+ into NADH in the presence of (*R*)-β-hydroxybutyrate at 1, 2, 4, 10, 20, 40, 60 mM concentration. The reactions were performed as described in the "*Experimental Procedures*" section. The curves were plotted employing Origin 2020 software (OriginLab, USA) and nonlinear regression analysis.

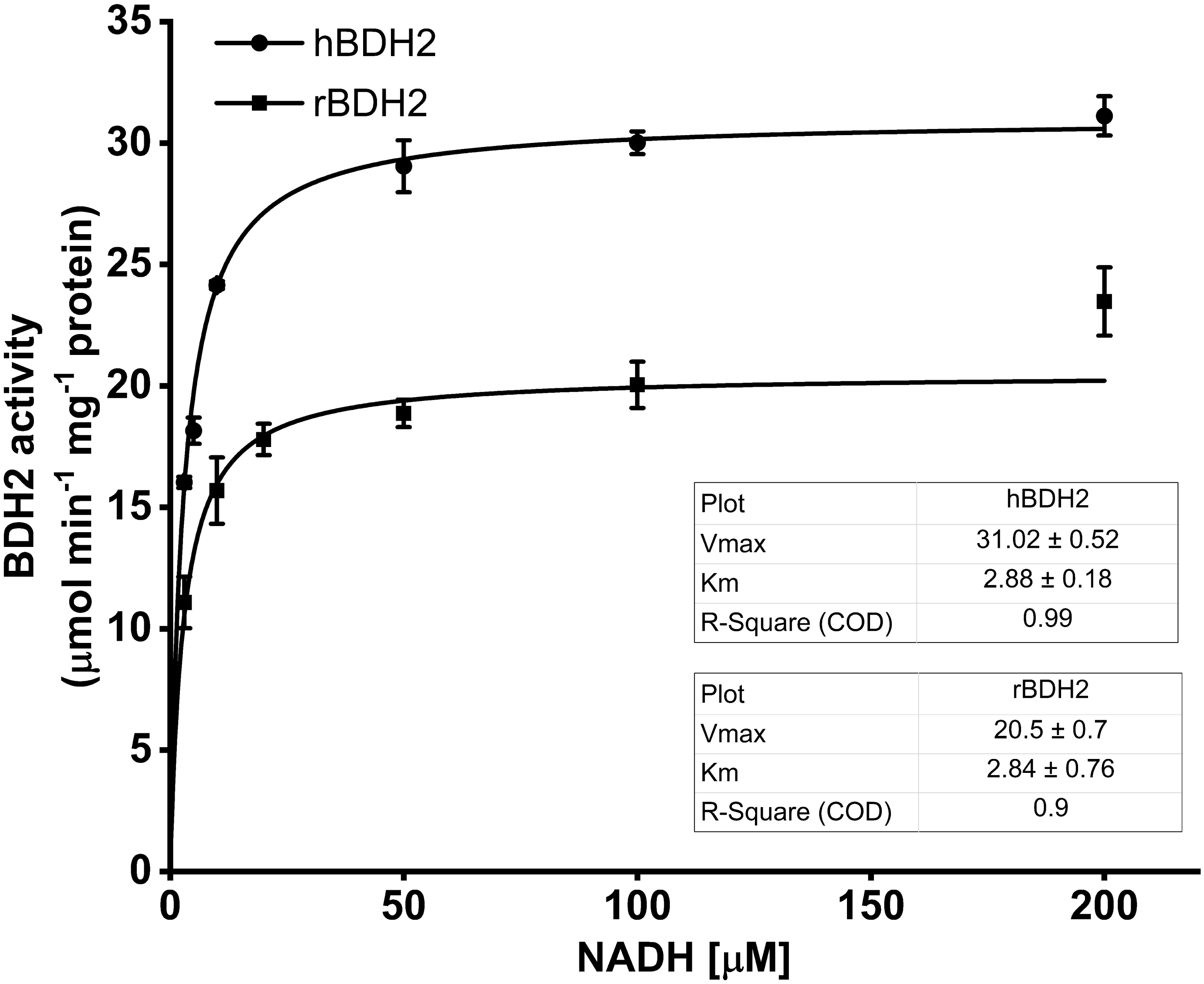

Fig. S3. Michaelis-Menten equation curves showing the effect of increasing concentrations of NADH on the reaction catalyzed by human and rat BDH2. The activity of human (hBDH2) and rat (rBDH2) enzymes (0.15 μg protein) was followed spectrophotometrically by measuring the conversion of NADH into NAD^+ in the presence of 2 mM 4-oxo-L-proline and NADH at 3, 5, 10, 50, 100, and 200 μM concentration. The reactions were performed as described in the "*Experimental Procedures*" section. The curves were plotted employing Origin 2020 software (OriginLab, USA) and nonlinear regression analysis.

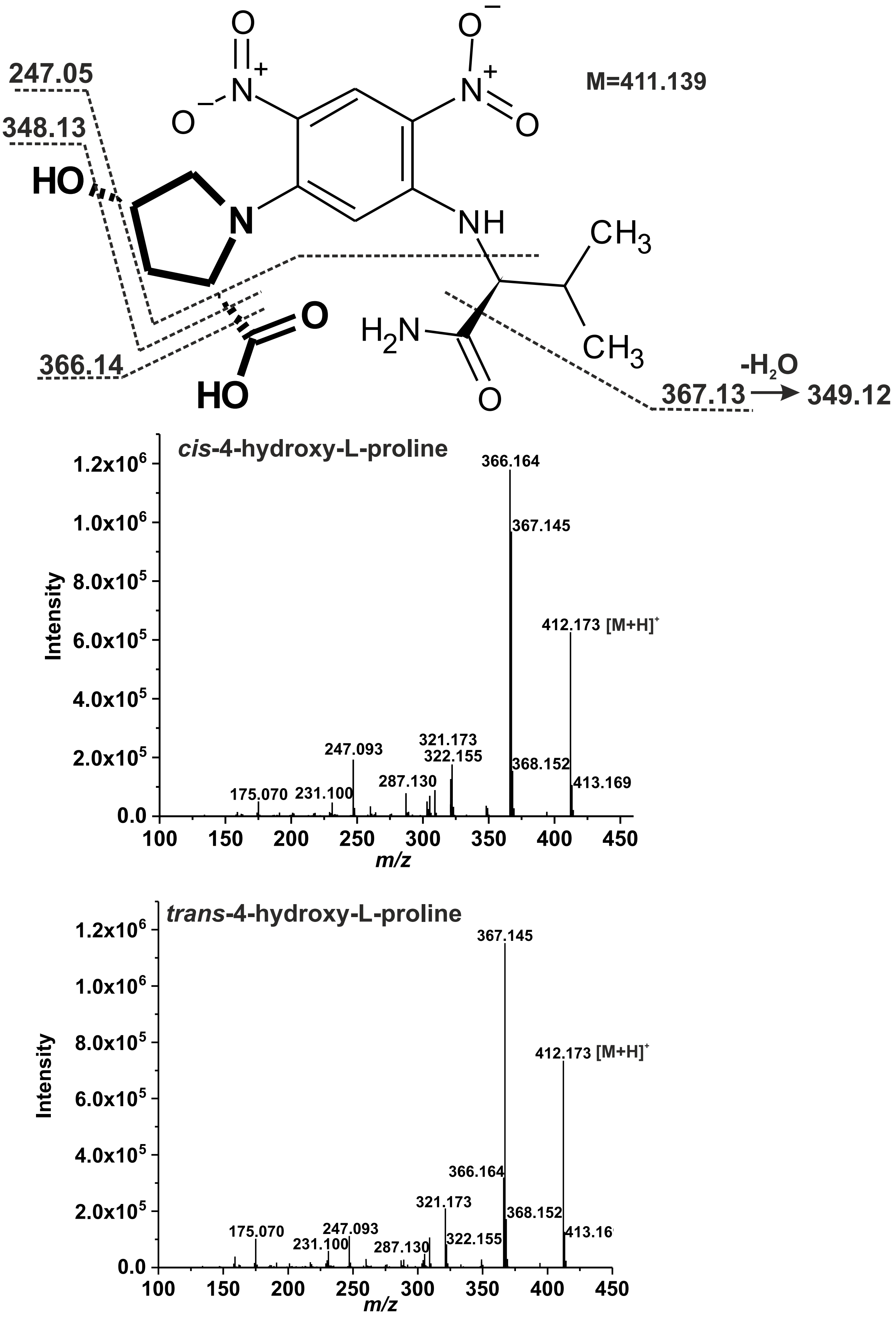

Fig. S4. Comparison of Q-TOF fragmentation spectra of L-FDVA derivatives of *cis*- and *trans*-4-hydroxy-L-proline. The commercial *cis*-4-hydroxy-L-proline and *trans*-4-hydroxy-L-proline were derivatized with L-FDVA, chromatographed on a reversed-phase C18 column, and analyzed by mass spectrometry. Mass spectra, covering the mass range m/z 100-450, were acquired. The structure of the L-FDVA derivative of *cis*-4-hydroxy-L-proline (in bold) and the assignments of some of its fragment ions are also shown.

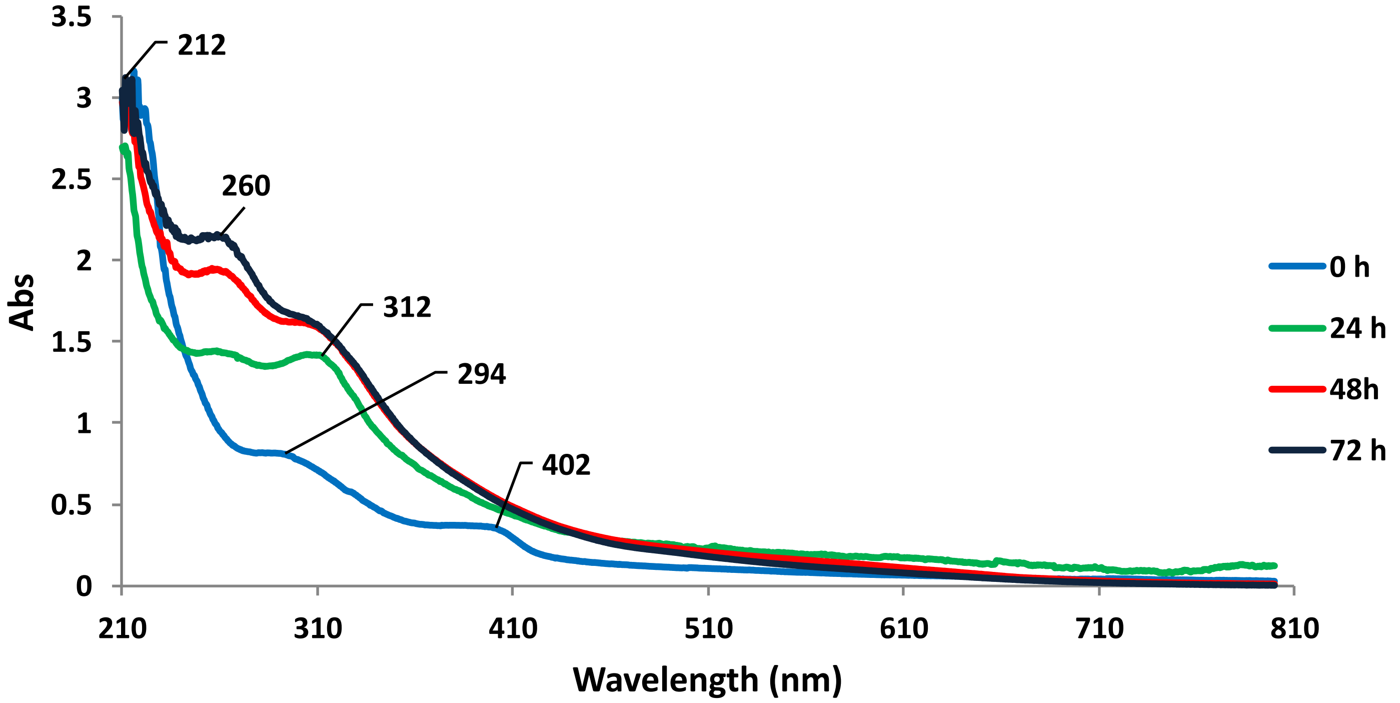

Fig. S5. Changes in absorption spectra (UV/Vis) of 4-oxo-L-proline solution during its incubation in the conditions used for culturing the cells.
Twenty millimolar 4-oxo-L-proline in H_2O (pH ≈ 7.0, adjusted with 1M $NaHCO_3$) was 2-fold diluted with 80 mM NH_4HCO_3, sterilized by filtration (0.2 μm), and incubated in aseptic conditions at 37°C for 72 hours. The absorption spectra (λ=210-800 nm) were recorded against 40 mM NH_4HCO_3 using Varian Cary® 50 UV spectrophotometer at 0, 24, 48, and 72 h of incubation. Samples collected at 24, 48 and 72 h were **10-fold diluted** with 40 mM NH_4HCO_3 prior to their measurement.

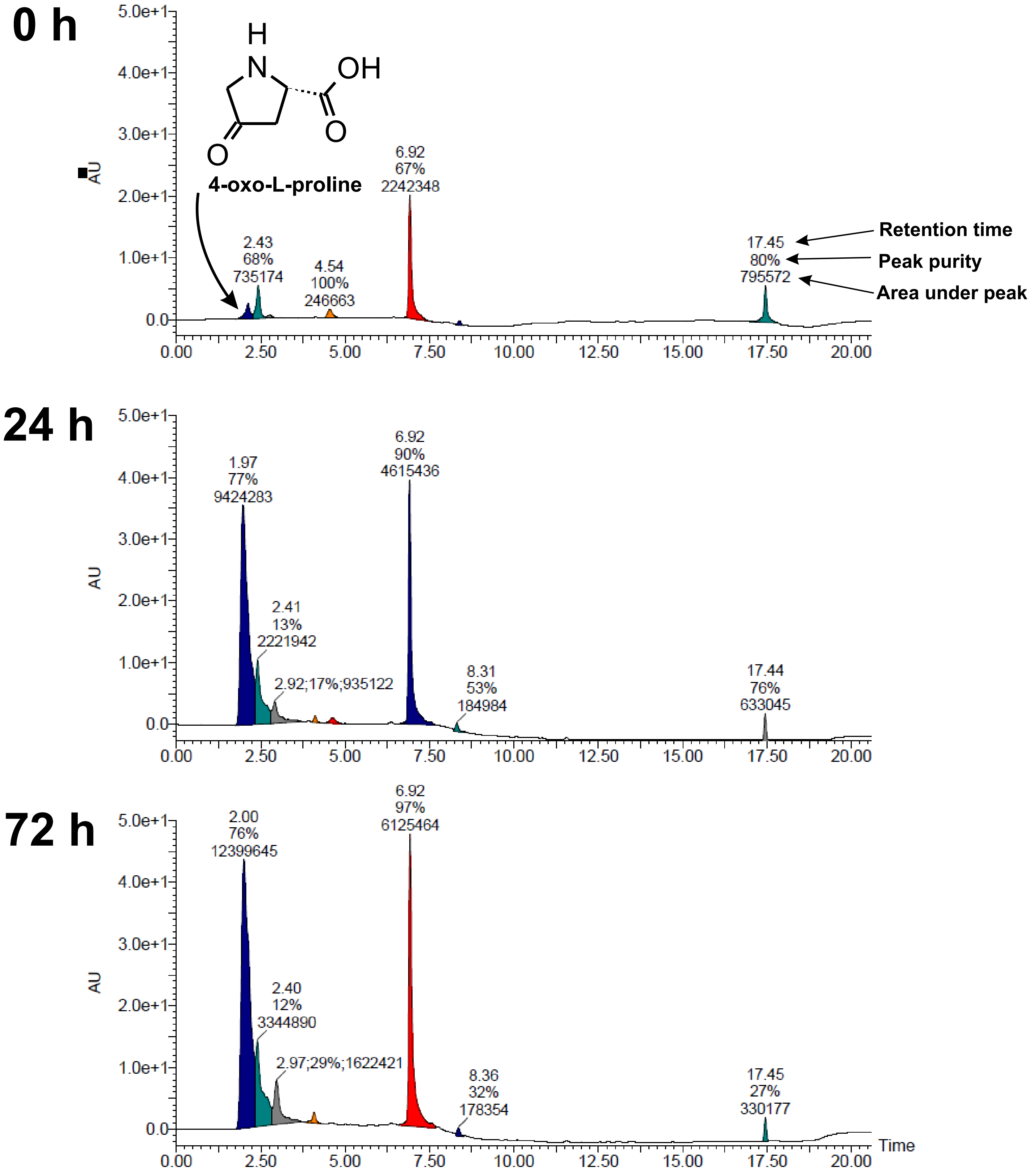

Fig. S6. RP-HPLC-PDA chromatograms (cumulative PDA absorbance for λ=220–700 nm) showing changes in the composition of 4-oxo-L-proline solution during its incubation in the conditions used for culturing the cells. Ten millimolar 4-oxo-L-proline in 40 mM NH_4HCO_3 was sterilized by filtration (0.2 µm), and incubated in aseptic conditions at 37°C for 72 hours. The samples were withdrawn at 0, 24, and 72 h of incubation. Ten microliters of each sample was separated in a gradient mode on the Zorbax SB-C18 column (ODS, 4.6 × 250 mm, 5-µm particle size) using Acquity UPLC (Waters) system equipped with Acquity UPLC PDA detector and Synapt G2 HDMS Q-TOF mass spectrometer fitted with an electrospray source. Mobile phases consisted of solvent A, containing 40 mM NH_4HCO_3 in the water, and solvent B, containing acetonitrile. The separation was performed in a linear gradient from 2 to 100% of solvent B for 16 min.
The presence of 4-oxo-L-proline in the indicated peak was verified by Q-TOF analysis. The peak purity values were calculated based on spectral data using MassLynx software (Waters).

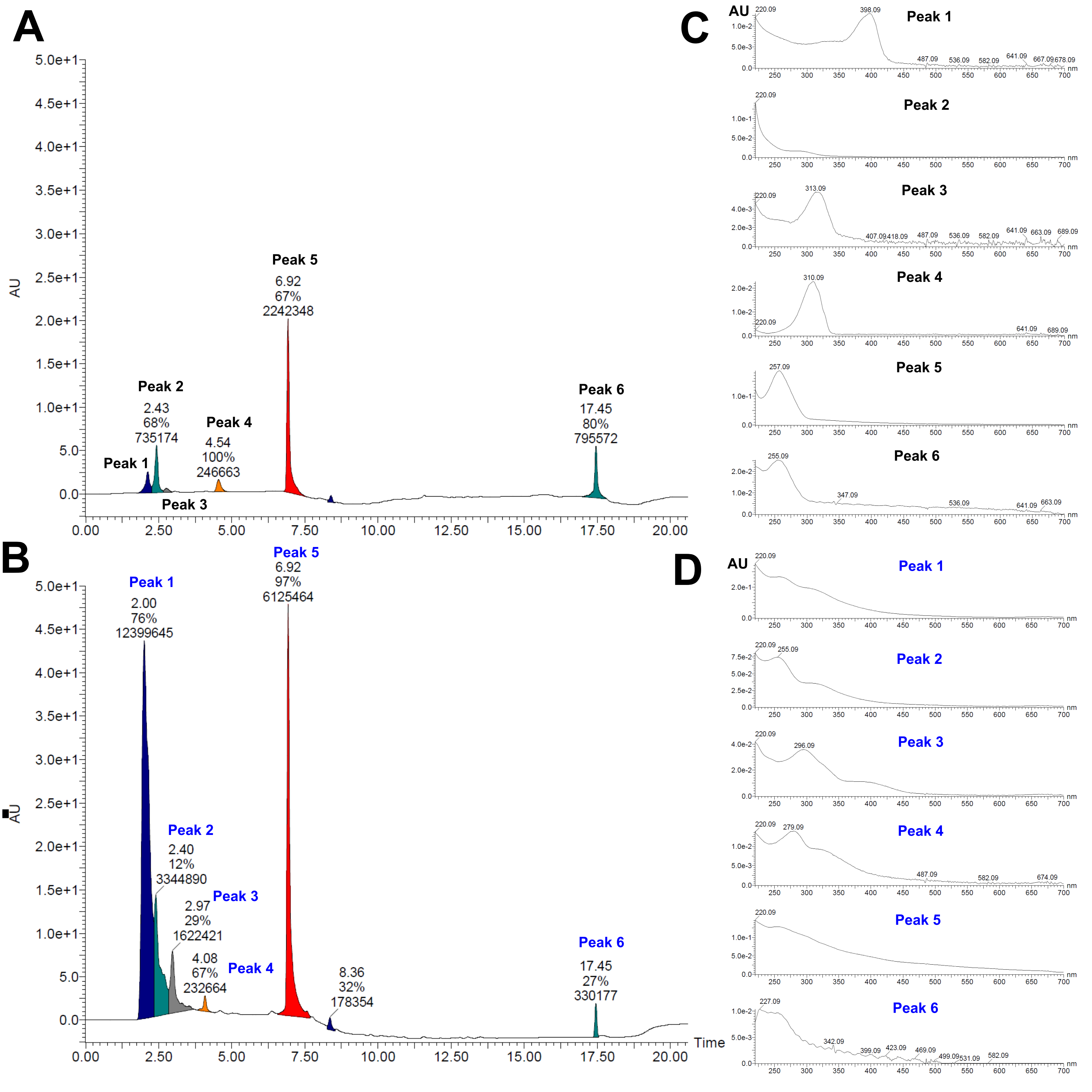

Fig. S7. RP-HPLC-PDA chromatograms (cumulative PDA absorbance for λ=220–700 nm) showing the time-dependent changes in the composition of 4-oxo-L-proline solution and UV-vis spectra of compounds present in selected peaks. Ten millimolar 4-oxo-L-proline in 40 mM NH_4HCO_3 was sterilized by filtration (0.2 μm) and incubated in aseptic conditions at 37°C for 72 hours. The chromatography of samples withdrawn at **A.** 0 and **B.** 72 h of incubation was performed as described in the legend to Fig. S6. **C.** and **D.** UV-vis spectra of compounds present in the peaks shown in chromatograms A. and B., respectively.

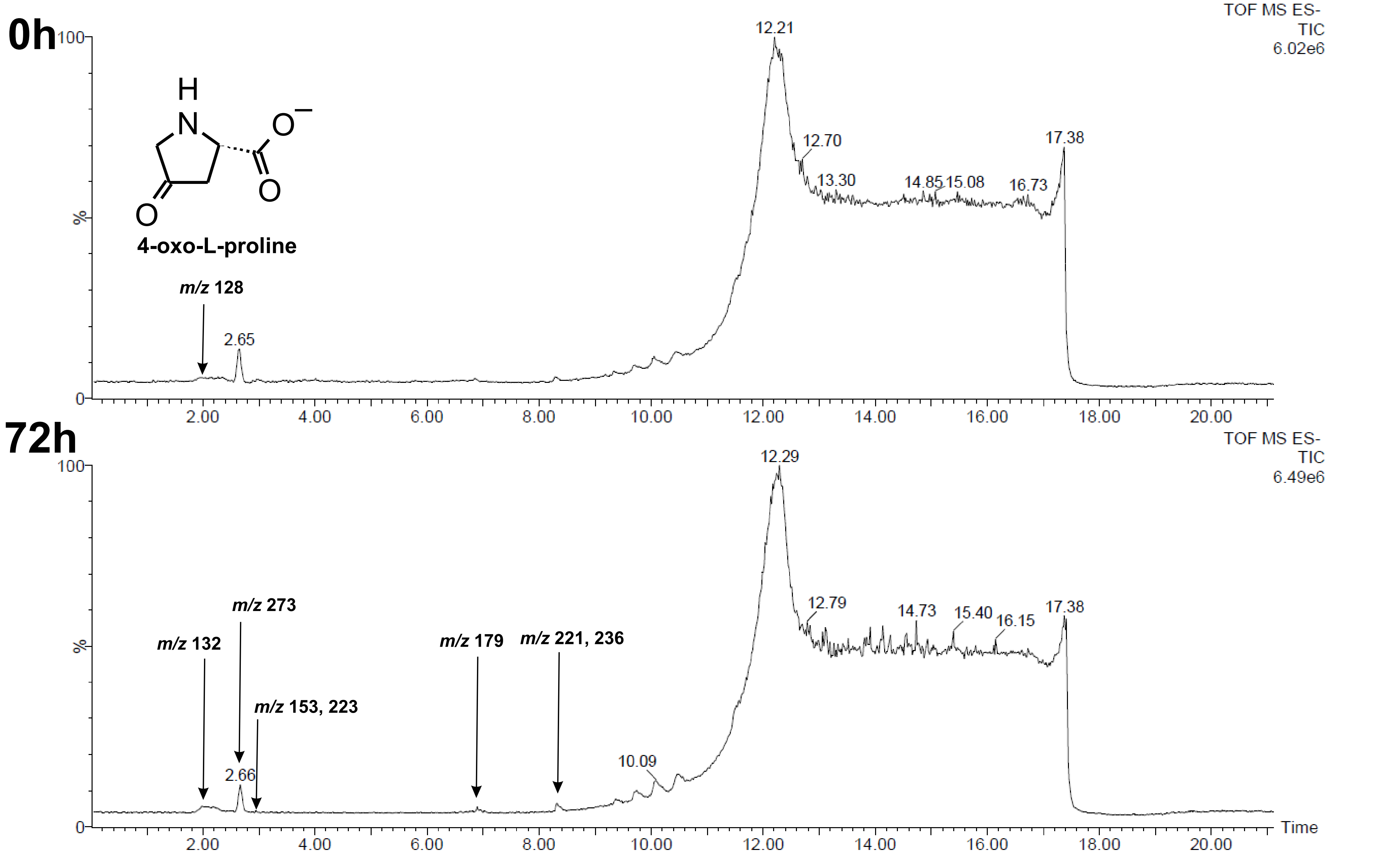

Fig. S8. RP-HPLC-Q-TOF chromatograms (total ion current for m/z 100–1200) showing the time-dependent changes in the composition of 4-oxo-L-proline solution. Ten millimolar 4-oxo-L-proline in 40 mM NH_4HCO_3 was sterilized by filtration (0.2 µm) and incubated in aseptic conditions at 37°C for 72 hours. The samples were withdrawn at 0 and 72 h of incubation. The chromatography was performed as described in the legend to Fig. S6. The eluate from the PDA detector was directed to Waters Synapt G2 HDMS Q-TOF mass spectrometer, operating in negative electrospray ionization-MS mode. The mass spectral data were recorded for m/z = 100-1200. The electrospray ionization source was set at a temperature of 90°C, the capillary voltage of 3.0 kV, and the cone voltage of 60 V. The flow rate of the desolvation gas (nitrogen) was 1000 liters/h, and the desolvation temperature was 150°C.

The *m/z* values are shown for single charged negative ions that were detected exclusively in 4-oxo-L-proline solution after 72h incubation.

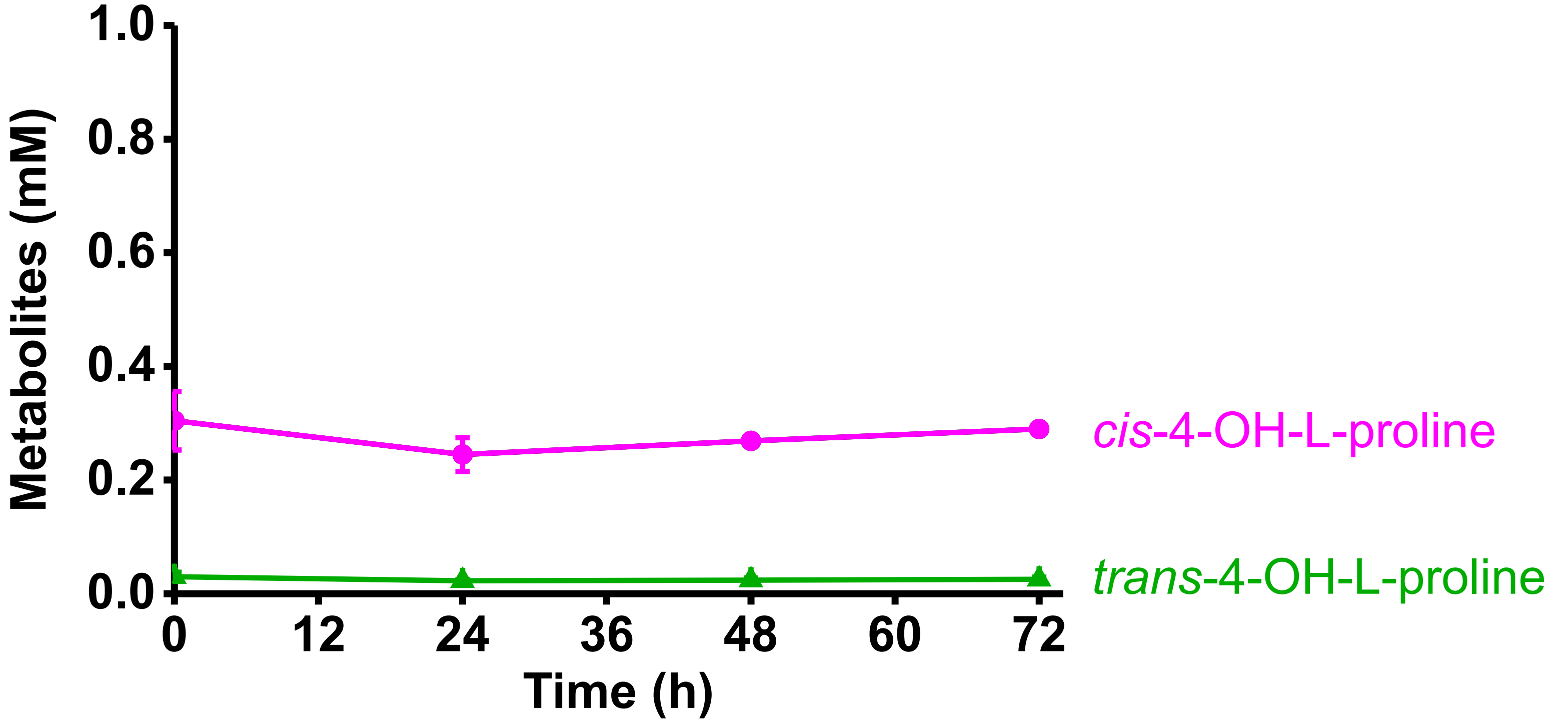

Fig. S9. Metabolism of *cis*-4-hydroxy-L-proline in wild-type HEK293T cells. The cells were plated in 6-well dishes and grown for 24 h. After that time, the cell culture medium was supplemented with 0.3 mM *cis*-4-hydroxy-L-proline (0 h time point), and the incubation was continued for up to 72 h as described under "*Experimental Procedures*" in the main text. Values are the means ± S.D. (error bars) of three independent experiments performed with cells from three different culture passages (n = 3). When no error bar is shown, the error is smaller than the width of the line.
